# Time-dependent antagonist-agonist switching in receptor tyrosine kinase-mediated signaling

**DOI:** 10.1186/s12859-019-2816-3

**Published:** 2019-05-15

**Authors:** Alokendra Ghosh, Ravi Radhakrishnan

**Affiliations:** 1 Department of Chemical and Biomolecular Engineering, University of Pennsylvania, Philadelphia, USA; 2 Department of Bioengineering, University of Pennsylvania, Philadelphia, USA

**Keywords:** RTK signaling, JAK-STAT pathway, Time-dependent switch, Global sensitivity

## Abstract

**Background:**

ErbB4/HER4 is a unique member of the ErbB family of receptor tyrosine kinases concerning its activation of anti-proliferative JAK2-STAT5 pathway when stimulated by ligand Neuregulin (NRG). Activation of this pathway leads to expression of genes like β-casein which promote cell differentiation. Recent experimental studies on mouse HC11 mammary epithelial cells stimulated by ligand Neuregulin (NRG) showed a time-dependent switching behavior in the β-casein expression. This behavior cannot be explained using currently available mechanistic models of the JAK-STAT pathway. We constructed an improved mechanistic model which introduces two crucial modifications to the canonical HER4-JAK2-STAT5 pathway based on literature findings. These modifications include competitive HER4 heterodimerization with other members of the ErbB family and a slower JAK2 independent activation STAT5 through HER4. We also performed global sensitivity analysis on the model to test the robustness of the predictions and parameter combinations that are sensitive to the outcome.

**Results:**

Our model was able to reproduce the time-dependent switching behavior of β-casein and also establish that the modifications mentioned above to the canonical JAK-STAT pathway are necessary to reproduce this behavior. The sensitivity studies show that the competitive HER4 heterodimerization reactions have a profound impact on the sensitivity of the pathway to NRG stimulation, while the slower JAK2-independent pathway is necessary for the late stage promotion of β-casein mRNA transcription. The difference in the time scales of the JAK-dependent and JAK-independent pathways was found to be the main contributing factor to the time-dependent switch. The transport rates controlling activated STAT5 dimer nuclear import and β-casein mRNA export to cytoplasm affected the time delay between NRG stimulation and peak β-casein mRNA activity.

**Conclusion:**

This study highlights the effect of competitive and parallel reaction pathways on both short and long-term dynamics of receptor-mediated signaling. It provides robust and testable predictions of the dynamical behavior of the HER4 mediated JAK-STAT pathway which could be useful in designing treatments for various cancers where this pathway is activated/altered.

**Electronic supplementary material:**

The online version of this article (10.1186/s12859-019-2816-3) contains supplementary material, which is available to authorized users.

## Background

ErbB4/HER4 belongs to the ErbB receptor tyrosine kinase family (consisting of EGFR, ErbB2, ErbB3, and ErbB4). These receptors regulate several critical signaling pathways that are frequently altered in cancers of lung, breast, prostate, etc. When activated by specific growth factors, they initiate multiple signaling cascades leading to the transcription of genes responsible for the determination of cell fate such as proliferation, differentiation or apoptosis [1]. Overexpression of these receptors or development of domain-specific mutations that allow them to be constitutively activated, causes them to promote various important pathways that drive the cell towards a program of proliferation and suppresses those that lead to apoptosis (cell death) or growth arrest through cell senescence. Compared to the other members of the ErbB family, the role of HER4 is incompletely understood [2]. In part, this has to do with the fact that this receptor has several unique features compared to other members of the family. It has four structurally and functionally different isoforms generated by mRNA splicing. Some of these isoforms undergo shedding of their ectodomain by proteolytic cleavage reaction mediated by Tumor Necrosis Factor-alpha converting enzyme (TACE). The remaining 80 kDa fragment is further cleaved by enzyme γ-secretase and is transported to the nucleus and other parts of the cell thereby taking part in critical cellular reactions that affect cell fate [3]. Unlike the other members of the ErbB family, HER4 has an anti-proliferative role through its activation of the JAK2-STAT5 pathway. Ligand Neuregulin (NRG), which is one of the ten natural ErbB family ligands and is often expressed in human tumors, can bind to and activate HER4. Upon activation and binding with JAK2, activated HER4 s80 domain can activate Signal Transducer and Activator of Transcription 5 (STAT5) causing it to translocate to the nucleus and initiate transcription of genes that promote differentiation [2, 3]. Hence it is essential to gain a mechanistic understanding of this pathway, particularly its dynamical behavior in response to NRG. Constructing and solving systems of nonlinear ordinary differential equations is a typical approach of modeling these pathways. The models are constructed based on experimental measurements of protein activities and rate constants and are used to predict the time-dependent dynamics of target gene/protein expression for different ligand stimulations. The predictions are validated through experimental measurements of the time-dependent protein activity through Western Blots or Immunoprecipitation studies [4]. Such an approach has been used for HER4 mediated JAK-STAT pathway before [5, 6]. However, these models failed to reproduce some interesting recent observations from experiments conducted in HC11 mouse mammary epithelial cell lines which prompted this computational study. In the experiments, it was observed that HER4 mediated transcription of anti-proliferative genes like β-casein milk genes follows a time-dependent switching behavior which cannot be explained by the previously published models. Here we propose an improved model of the HER4-JAK-STAT pathway that incorporates additional interactions which have been previously reported in the literature but were not part of the original models. Some of these reactions include competitive heterodimerization of HER4 receptors with other members of ErbB family and JAK2 independent activation of HER4. The latter phenomenon of signaling through parallel pathways has some similarities with signaling pathways driven by constitutively activated mutant forms of EGFR [7]. Identification of such pathways can be critical to gain a better understanding of the HER4 induced JAK/STAT pathway and leverage its anticancer role by designing an appropriate treatment strategy to treat cancers in cell lines where this receptor is significantly expressed.

## Results

### HER4 signaling through JAK-dependent pathway

The computational study was motivated by some intriguing results from experiments conducted on HC11 mouse mammary epithelial cells [8] stimulated with different ligands including NRG. In these studies, HC-11 cells were grown and maintained at 5% CO2 in complete medium (RPMI 1640, 10% FBS, 2 mM L-glutamine, 100 U/mL penicillin, 100 μg/mL streptomycin, 1 μg/mL hydrocortisone, 10 ng/mL murine EGF and 5 μg/mL insulin). Cells were seeded in 6-well plates at a density of 2 × 104 cells/cm^2^ and allowed to grow to 100% confluence to induce differentiation. The cells were maintained at confluence for 2 days in serum-free/EGF-free medium to induce competence. The competent cells were then stimulated with NRG to induce differentiation. Three stages of the differentiation process were identified: Stage 1 refers to growing the cells to confluence, Stage 2 refers to maintaining the cells at confluence for 48 hours to induce a state of competence, and Stage 3 refers to cell stimulation with HER4 ligand. RT-PCR and ELISA based Transcription Factor activation assay were performed in differentiating HC11 cells to characterize the signaling dynamics in the HER4-mediated JAK/STAT pathway. Specifically, the following read-outs of HC11 mammary differentiation were assayed:

- Levels of activated STAT5A and glucocorticoid receptor (GR) in the nucleus
- Expression of the β-casein milk gene mRNA.

STAT5 and GR are the two key transcription factors which are both activated during HC11 cell differentiation and synergize on the β-casein gene promoter [9]. In the experimental studies, the steroid hydrocortisone (HC), which signals through GR was included in the medium. It was shown that in cells stimulated with NRG in the absence of HC in the medium, no β-casein was expressed. However, upon addition of HC, HER4 induced robust expression of β-casein. HC was required in Stage 2 of the differentiation process as well as Stage 3. Hence the β-casein expressions obtained by varying NRG levels were normalized by the control case of only HC present in the medium.

**Fig. 1:**
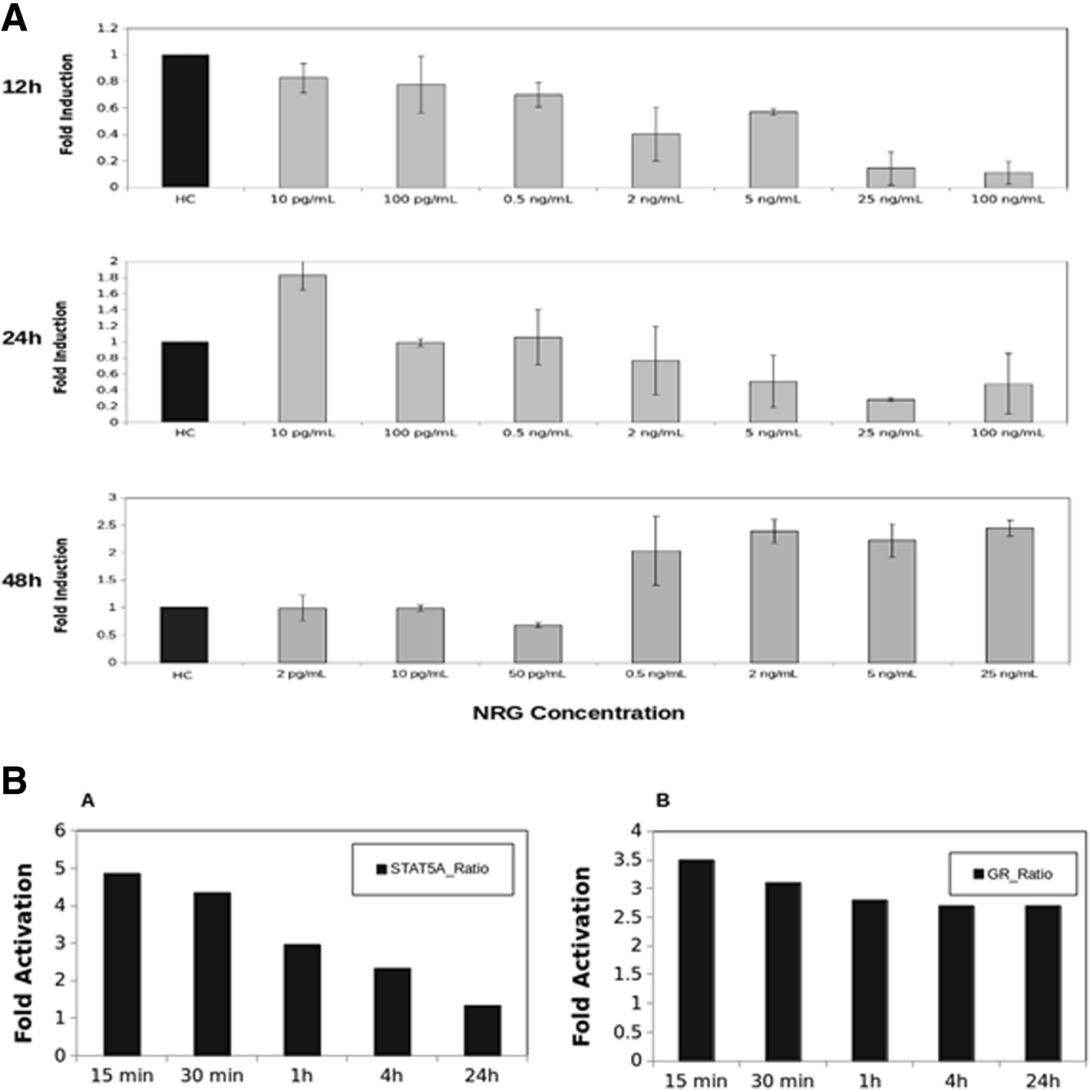
Experimental data from HC-11 murine mammary epithelial cell lines (reproduced with permission from Telesco et al. [8]). **a** Shows the RT-PCR results of the expression of the differentiation marker β-casein gene at three different time intervals (0–12, 12–24 and 24–48 h) for varying levels of ligand NRG stimulation. The results were compared with β-casein expression with Hydrocortisone (HC) as a control. **b** Shows the ELISA TF activation assays of the transcription factors STAT5 and Glucocorticoid Receptor (GR) at the nucleus at different time points post ligand stimulation

In tracking the response of the HC11 cells to HER4 stimulation by NRG in the presence of HC at various time points, it was observed (Fig. 1) that at early time points post-stimulation, NRG does not exhibit any effect on β-casein expression. However, at 12-h post-stimulation, it was found that increased stimulation with NRG decreased levels of β-casein, relative to the control (hydrocortisone). At 24–48 h post-stimulation, NRG began to increase β-casein expression levels at sufficiently high ligand concentrations. This result has not been discovered previously, as the few studies examining the HER4-STAT5A pathway focused mostly on gene expression at earlier time points post stimulation [9, 10].

**Fig. 2:**
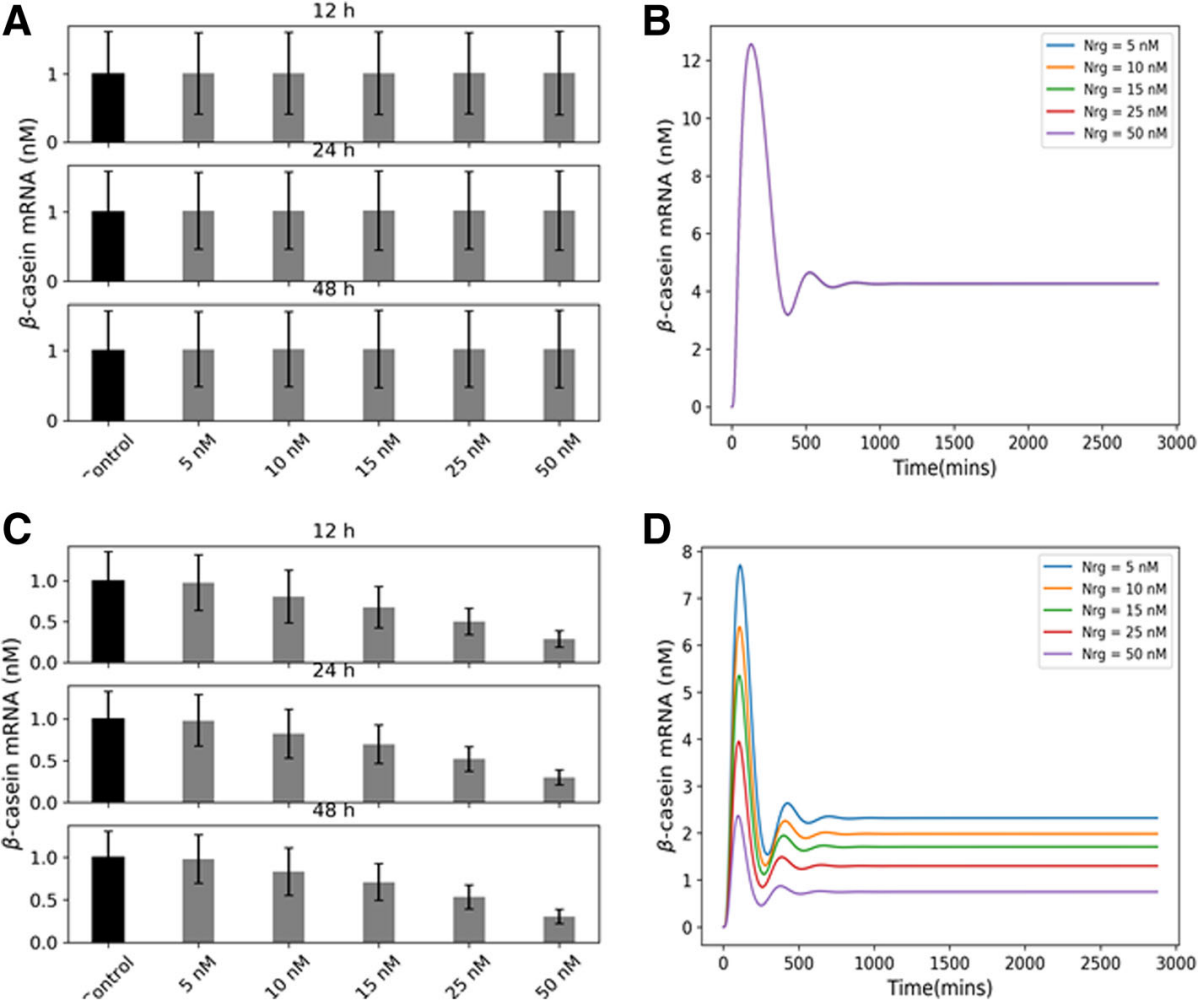
β-casein expressions predicted by the literature model and a modified version with competitive HER4 heterodimerization. Panels **a** and **b** show the time-integrated β-casein values (obtained by integrating the solutions of the nonlinear system of ODE at different time points over different time intervals - 12,24 and 48 h in three separate subpanels). The panels also show the instantaneous values (obtained by solving the nonlinear systems of ODEs at different time points) respectively as predicted by the literature models of JAK-STAT pathway for varying levels of NRG stimulations. Panels **c** and **d** show the same time-integrated and instantaneous levels of β-casein when competitive HER4 heterodimerization is included in the model. The time-integrated plot contains three subpanels showing the expression at three different time points (12, 24 and 48 h). The error bars are obtained by generating an ensemble of models by sampling over parameter space. The instantaneous plots are for 0–48 h range at five different NRG stimulations

**Fig. 3:**
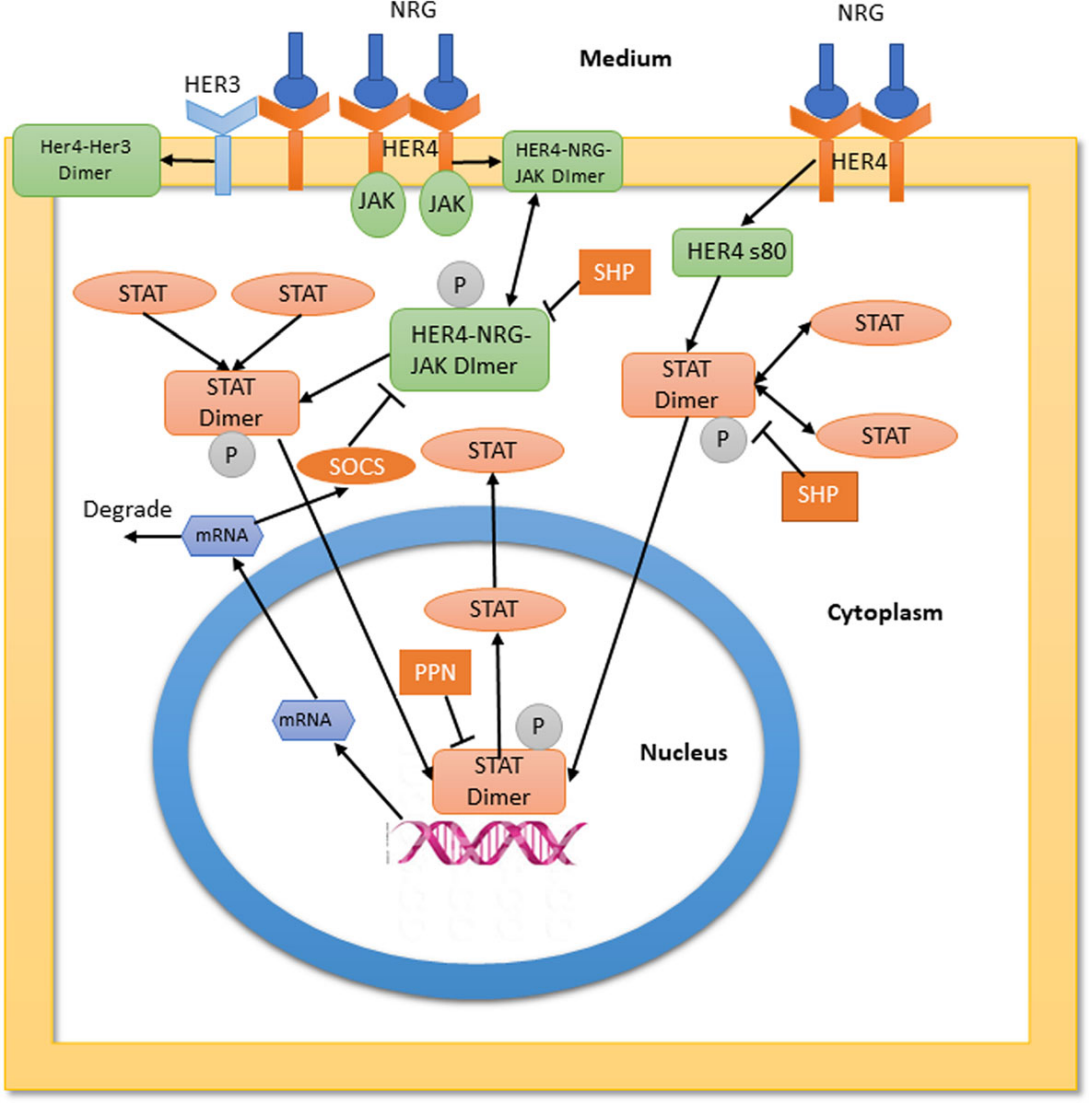
Schematic diagram of the HER4-JAK2-STAT5 pathway modeled in this paper. More details are available in the Methods section. We introduced cytoplasmic and nuclear compartments and transport reactions between the compartments as indicated. The heterodimerization reactions are indicated with a side branch from HER4 and not shown explicitly

**Fig. 4:**
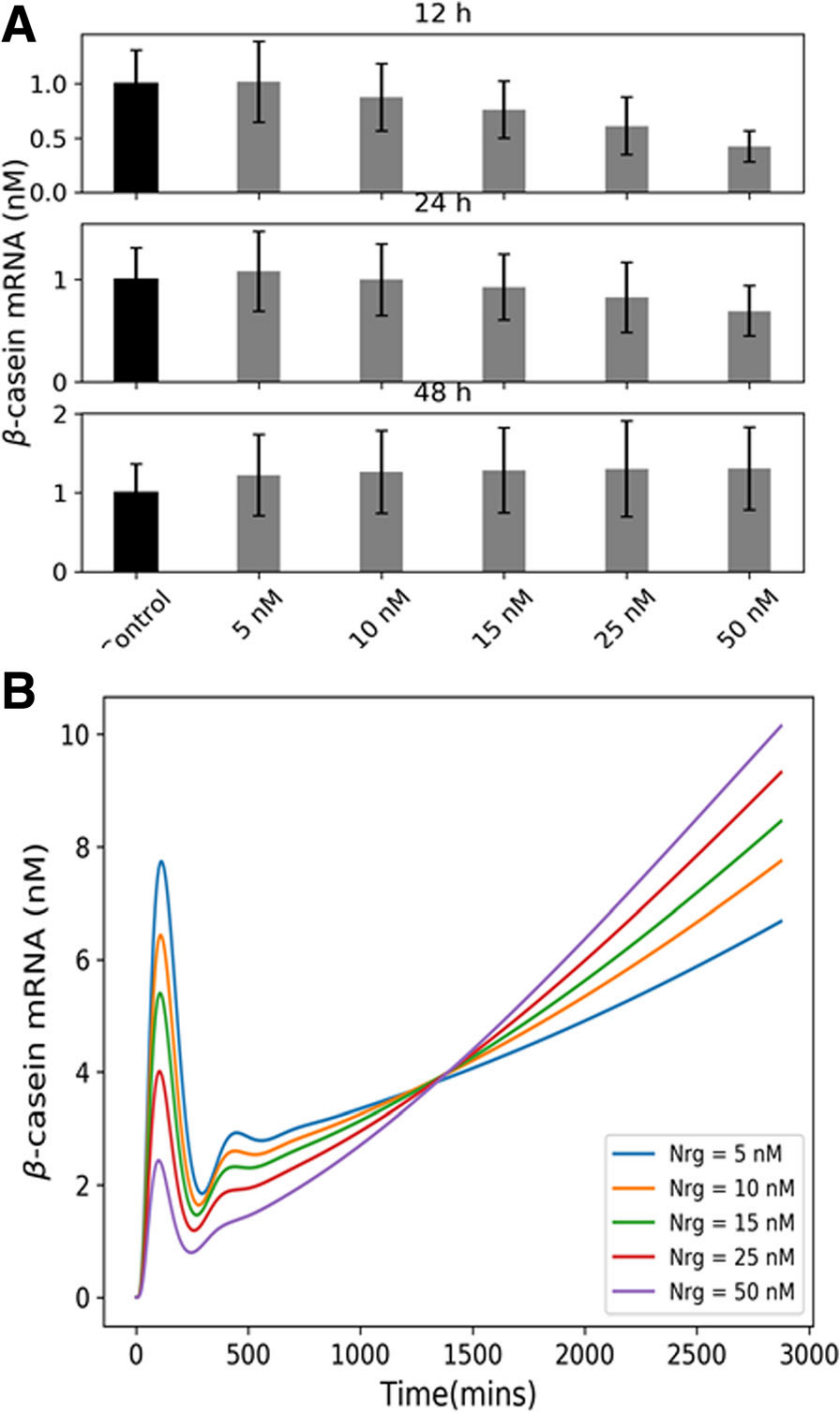
β-casein expressions for a model with competitive heterodimerization and parallel JAK-independent activation of STAT5. Panels **a** and **b** show the time integrated and instantaneous levels of β-casein for this model. The time-integrated plot contains three subpanels showing the expression at three different time points (12, 24 and 48 h). The error bars are obtained by generating an ensemble of models by sampling over parameter space. The instantaneous plots are for 0–48 h range at five different NRG stimulations

**Fig. 5:**
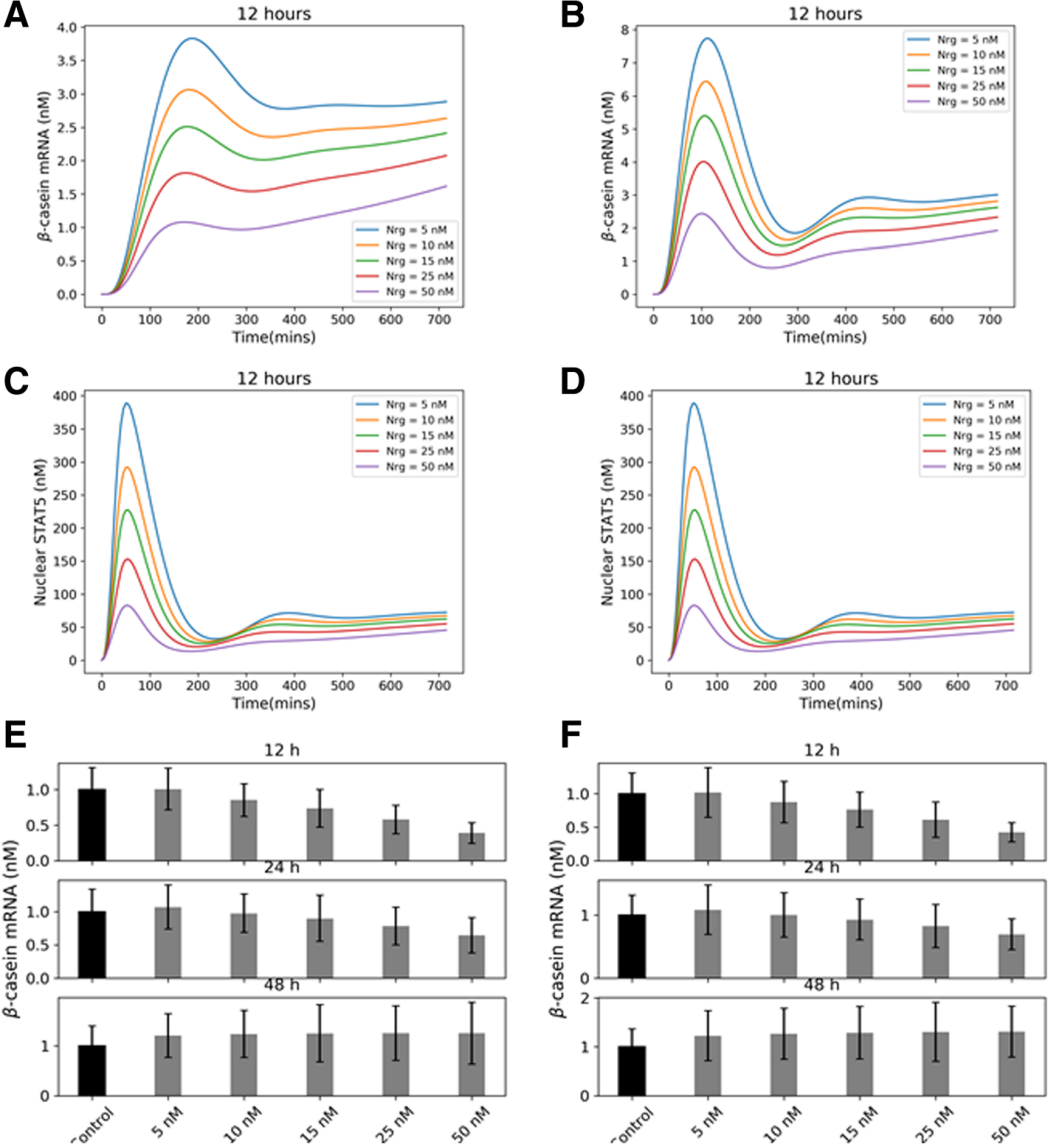
Effect of the mRNA transport rates on the transcription time delay. **a** and **b** show the β-casein mRNA expression time profiles at low and baseline values of the rate of mRNA export. Plots in **c** and **d** show the corresponding nuclear STAT5 levels. By decreasing the transport rate ten fold, we can see almost 3 h delay between STAT5 and β-casein mRNA peaks, showing that these rates contribute to a large extent towards the observed time delay in experiments. **e** and **f** show the integrated β-casein levels at the three time points which shows that the temporal switch is preserved at low mRNA transport rates. The error bars are obtained by generating an ensemble of models by sampling over parameter space

As a first step towards understanding the mechanism of this time-dependent switching behavior observed in the experiments, we used mathematical models of JAK-STAT pathways reported before in literature [5, 11]. In these models, HER4 is activated in a JAK2-dependent fashion, and the HER4-JAK2 dimer activates cytoplasmic STAT5 which dimerizes and translocates to the nucleus. This model was run with increasing levels of ligand (NRG) stimulation in the same range as used in the experiments [8]. Although these models were developed for shorter time ranges of 0–12 h we wanted to see to what extent this model can capture the early time behavior observed in experiments. In Fig. 2a and b, we plot the time-integrated β-casein gene expression as a function of ligand NRG stimulation and instantaneous time profile of β-casein gene. To show how model parameter uncertainties influence its predictions we sampled the parameter space using Latin Hypercube Sampling (see Methods) after setting biologically proper bounds on each parameter. This sampling was used to generate an ensemble of model parameter values which were used to run the model and obtain a set of output values. The average value of this set is reported, and the average dispersion is shown in the form of error bars. All the time-integrated plots in Figs. 2, 3, 4 and 5 are generated using this procedure.

The plots corresponding to the literature model show little sensitivity towards NRG in terms of β-casein expression at all three time intervals. The literature model has several negative regulators including two cytoplasmic phosphatases for JAK2 and STAT5, one nuclear phosphatase for STAT5 and negative feedback through SOCS protein deactivating HER4-JAK complex. SOCS is another product of STAT5 mediated transcription apart from β-casein. The latter is expected to limit the response and prevent any increase in transcription due to higher ligand stimulation. However, the insensitivity of β-casein towards NRG is inconsistent with the observations from the experiments. In the 0–12 h interval, the experiments show a decrease in the β-casein gene expression with increasing ligand stimulation. This observation suggested that there are additional mechanisms which are not considered in the canonical JAK-STAT pathways published earlier in the literature.

### Competitive heterodimerization involving HER4 upon ligand binding

It has been previously reported in the literature that competitive heterodimerization of HER4 with other members of ErbB family can result in a decrease in HER4 activity for a particular reaction pathway with increasing ligand stimulation (so-called partial agonists) [12–15]. To explore this effect, we introduced additional heterodimerization reactions of HER4 with ErbB3. Introduction of such reactions resulted in a decrease in the β-casein activity with increasing NRG stimulation consistent with experimental observations. The reduction is evident from Fig. 2c and d where we have again plotted both integrated and instantaneous β-casein mRNA concentrations. Since there are several competing reactions possible for a fixed number of free HER4 receptors at the cell surface, increasing ligand stimulation promotes the more favorable (heterodimerization) reaction at the expense of the less favorable (HER4-JAK dimerization). Addition of competitive heterodimerization, however, does not increase β-casein activity in the 24–48-h interval as observed in the experiments. This observation suggests additional reactions are at play during this late period contributing to an increase in the β-casein gene expression.

### Combined model with JAK-dependent and JAK-independent HER4 signaling

It has been reported before in the literature [16, 17] that HER4 can activate STAT5 independent of JAK2 although with lower rates compared to the canonical JAK-dependent pathway. We hypothesized that such a slower JAK-independent pathway might likely become active in the later stages (24–48 h) where it can contribute to an increase in gene expression with increasing ligand stimulation after the faster and more favorable heterodimerization reactions have equilibrated. Also, this pathway is not affected by negative feedback through SOCS, which can only act on the JAK-bound complex. We incorporated such a pathway in our model to explore late time behavior (see the method section for more details). The combined pathway is shown in the schematic diagram in Fig. 3 where the JAK-dependent and JAK-independent pathways are highlighted.

When this combined pathway was stimulated with increasing NRG stimulations (plotted in Fig. 4), we were able to reproduce the time-dependent switching behavior seen in the experiments: that is, in the 0–24 h period the β-casein activity decreases with ligand stimulation, and it increases in the later stages (24–48 h). From the β-casein mRNA time profiles, we see that after the 24-h mark there is a transition from negative to positive dependence of β-casein mRNA towards ligand stimulation. In this regime, the β-casein levels continue to increase.

### Time delay in β-casein mRNA transcription

In the HC-11 cell line experiments mentioned above additional ELISA-based transcription factor (TF) activation assays were also performed to measure the activity of STAT5A and Glucocorticoid receptor (GR) which are the two transcription factors necessary for transcription of β-casein mRNA. It was observed that even though STAT5A and GR activity was highest in the nucleus 15–30 min post ligand stimulation, a significant activity of these transcription factors persisted even 24 h post stimulation. These findings were consistent with previous ChIP-Seq studies [18, 19] which assayed for binding of various TFs (including STAT5A and GR) to the β-casein gene promoter at different time points following stimulation with prolactin (PRL). These studies showed that although several of the TFs assemble on the promoter at early time points, the RNA polymerase does not bind and commence transcription until 24 h post-stimulation.

To incorporate this delay in the transcription and detection of β-casein mRNA, we introduced two significant transport rates in our model — nuclear import of activated STAT5 dimer and nuclear export of β-casein mRNA. The plots of instantaneous β-casein mRNA and activated STAT5 dimer at the nucleus between 0 and 12 h time points for two different mRNA transport rates show that with low rates of mRNA export we get a significant delay in the mRNA peak activity. These results are consistent with previous experimental and modeling studies [5, 17] which identified these transport rates as essential determinants of the signaling activity of this pathway. Also, such delay in transcription did not affect the time-dependent switching behavior (Fig. 5e and f) in the β-casein expression which occurs over a much longer time scale.

Additional file 1: Figures S5-S14 explore the effect of the reaction parameters such as initial receptor numbers, phosphatase activity, transcription rates, HER4 homo, and heterodimerization rates and the rates of JAK-independent activation of STAT5 by HER4 on the β-casein expression in the 0–12, 12–24 and 24–48 h time intervals. These results complement the findings from the Global Sensitivity Analysis (next section).

### Global sensitivity analysis

To systematically explore the effect of model parameters on various output quantities of interest (such as β-casein expression, time-dependent switch in β-casein expression, β-casein transcription time delay, etc.), we performed sensitivity analysis. The HER4-JAK-STAT signaling network is a highly nonlinear system, and most of the parameters can vary over a wide range in the corresponding biological system. Hence, a Global Sensitivity Analysis (GSA) which considers the combined effect of the model parameters rather than one at a time is a more appropriate method here [20]. We use a particular type of global sensitivity analysis called Sobol Sensitivity Analysis [21] method for this study (details in the Methods section). The analysis procedure involves:

- Sampling over the space of model parameters to create an ensemble of models.
- Running the simulations for each member of the ensemble.
- Calculating the output quantity of interest from the results for each member of the ensemble.
- Determining the effect of the model parameter variation on the variation in the output quantity using Sobol Sensitivity Analysis.

The input parameters here are the initial concentrations of all the species and the kinetic rate constants of the reactions. Three main output quantities were calculated from the results obtained by running the ensemble of models, and the Sobol sensitivity coefficients were calculated for each of these three quantities. These are:

#### Integrated β-casein levels

β-casein mRNA levels integrated over the whole time interval (0–48 h) is a direct output of the model which we have used to present the results in the previous section. This quantity is an automatic choice for the sensitivity analysis.

#### Time-dependent switch in the β-casein expression

we consider the relative contributions to the time-integrated β-casein levels of the JAK-dependent and the JAK-independent parts of the pathway. Since we know from the above results that the JAK-dependent pathway is operational in the earlier part (0–12 h) while the JAK-independent pathway in the later part (24–48 h) we can calculate the integrated mRNA levels separately for these time intervals and calculate their ratio. When this ratio is near unity, it indicates equal contributions of both these pathways to the net β-casein expression. On the other hand, a significant value of this ratio will indicate that the main contribution comes from JAK-dependent pathway and the model does not show a time-dependent switch. A similar interpretation can be made when the ratio is small.

#### Transcription time delay

We get the delay between the times when activated STAT5 dimer reaches the nucleus and when β-casein mRNA transcription starts by getting the difference between the peak times of nuclear STAT5 dimer and cytoplasmic β-casein mRNA. The previous results also showed that the dynamical behavior of the combined pathway is much dependent on the level of ligand NRG stimulation. So, we decided to conduct the global sensitivity analysis at three different NRG stimulation – low (10 nM), medium (20 nM) and high (50 nM).

### Time-integrated β-casein levels

**Fig. 6:**
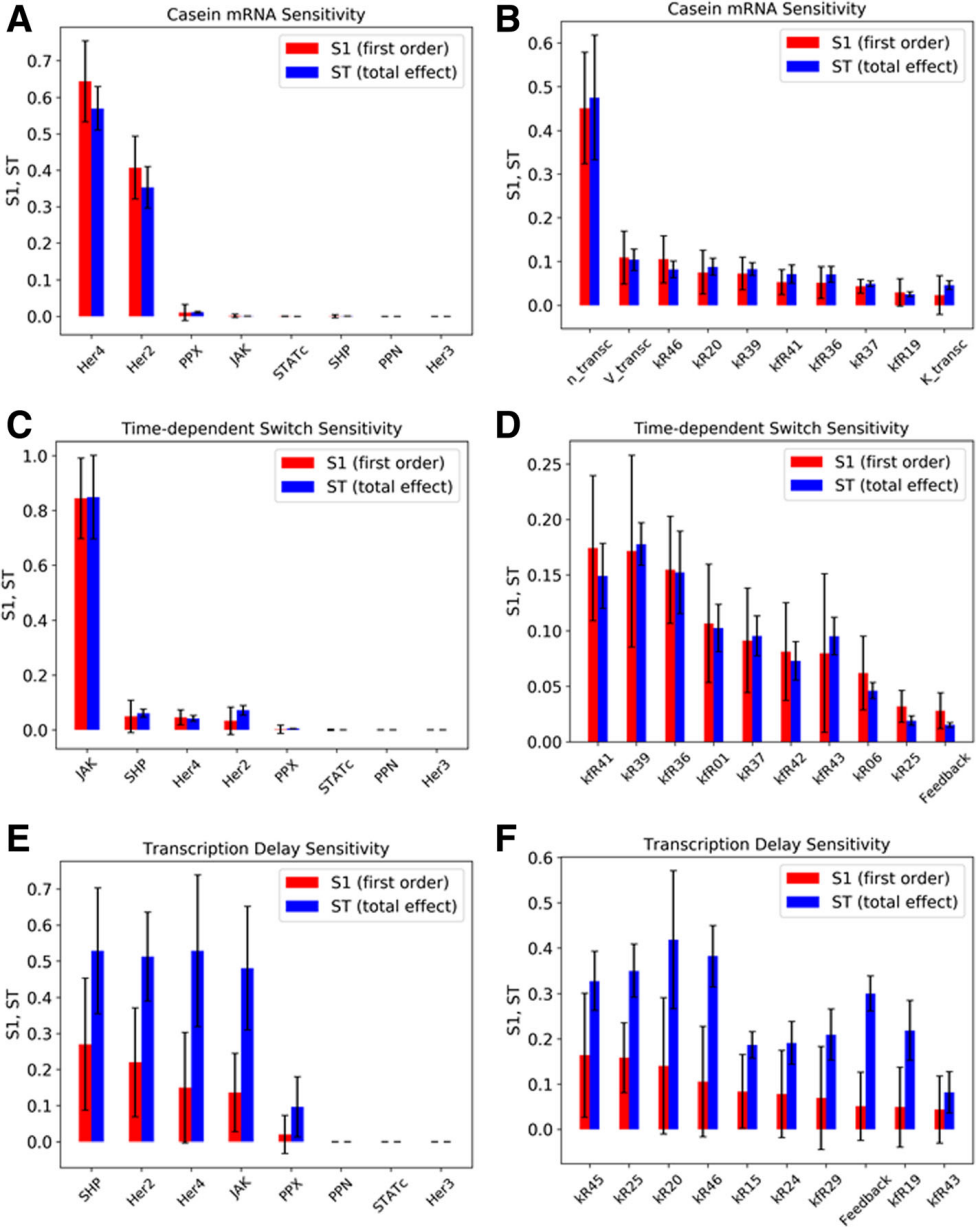
First order (S1) and total effect (ST) Sobol Sensitivity Coefficients for the most sensitive species and kinetic rate constants for different model outputs. See Methods section for the definition of these sensitivity coefficients. **a** and **b** show the sensitivities concerning the species and parameters for absolute β-casein levels as output. **c** and **d** show sensitivities concerning the ratio of contributions to β-casein transcription by the JAK-dependent and JAK-independent parts of the pathway. **e** and **f** show the sensitivities concerning the time delay between STAT5 import into the nucleus and β-casein transcription. A sensitivity coefficient ST > > S1 indicates significant higher order contributions to the sensitivity

We performed the global sensitivity analysis concerning the total integrated β-casein mRNA levels in the cytoplasm. The results are presented separately for the initial amounts of species and the kinetic rate constants (Fig. 6a and b). The plots show both the first order (S1) and the total effect sensitivities (ST) of the top 10 most sensitive species and parameters of the model. Table 1 below (left column) shows the names of the parameters and the reactions they belong to. From the results, we see the β-casein mRNA expression is sensitive to initial numbers of Her4 and Her2 receptors. It is also sensitive concerning the phosphatases SHP and PPX and JAK which are expected. As for the reactions the sensitivity is high for the transcription parameter (including the Hill coefficient), suggesting that the β-casein expression is sensitive to the mechanism of the transcription reaction. We want to find if the same holds when we consider our main output quantities of interests – time-dependent switch and transcription delay. Among other reaction parameters, we see that the rates of JAK-independent activation of STAT also features among the top sensitive parameters. The sensitivity plots also show that the total effect sensitivity *S*_*T*_ ≈ *S*_1_ here for all the sensitive parameters which suggests that there is not much higher order effect of parameters on the absolute β-casein levels.

**Table 1:** Table summarizes the top 10 most sensitive system parameters for the global sensitivity analysis concerning integrated β-casein (left), transcription time delay (middle) and time-dependent switch (right). For each output parameter, some of the hypothesized mechanisms are highlighted. For example, for transcription time delay the mRNA and STAT transport rates are in bold

Casein (JAK-independent in bold)		Delay (Transport Reactions in Bold)		Ratio (Heterodimerization reactions in bold)	
Parameter	Name	Parameter	Name	Parameter	Name
**n_transc**	**Transcription Hill Coefficient**	**kR45**	**mRNA Nuclear Export Casein**	**kfR41**	**HER4 Heterodimerization**
**V_transc**	**Transcription Half-Maximum rate**	kR25	Translation	kR39	STATc Phosphorylation JAK independent
kR46	mRNA degradation rate	kR20	Nuclear STAT dimer dephosphorylation	**kfR36**	**IFNR (HER4 s80) Formation**
kR20	Nuclear STAT dimer dephosphorylation	kR46	mRNA degradation rate	kfR01	HER4 JAK Binding
kR39	STATc Phosphorylation JAK independent	**kR15**	**STAT Dimer Nuclear Translocation**	**kR37**	**IFNR (HER4 s80) Activation**
kfR41	HER4 Heterodimerization	**kR24**	**mRNA Nuclear Export**	**kfR42**	**HER4 Heterodimerization Constitutive**
kfR36	IFNR (HER4 s80) Formation	kfR29	STATc SOCS HER4 JAK SHP Binding	**kfR43**	**HER4 Heterodimer Ligand Binding**
kR37	IFNR (HER4 s80) Activation	SOCS_Binding_Rate	SOCS Mediated negative feedback	kR06	STATc Phosphorylation JAK Dependent
kfR19	Nuclear STAT Dimer PPN Binding	kfR19	Nuclear STAT Dimer PPN Binding	kR25	Translation
**K_transc**	**Transcription Equilibrium Coefficient**	kfR43	HER4 Heterodimer Ligand Binding	SOCS_Binding_Rate	SOCS Mediated negative feedback

### Time-dependent switch in β-casein expression

The next output parameter we considered is the ratio of β-casein mRNA expression during the JAK-dependent pathway (0–12 h) to that of JAK-independent pathway (12–48 h). As explained previously, this quantity can be an indicator of whether and to what extent there is a time-dependent switch in β-casein mRNA expression. This output quantity was found (Fig. 6c and d) to be particularly sensitive to HER4 heterodimerization and homodimerization reactions (both ligand-dependent and constitutive) as well as the JAK-independent HER4 activation. The results confirm our hypothesis that both these additions to the literature JAK-dependent pathway are sufficient for producing a time-dependent switch. Interestingly the transcription reaction parameters which influenced the absolute time integrated mRNA levels to a high degree (from the previous section) did not appear in the list of top parameters for the time-dependent switch. These insights highlight the importance of selecting a proper output quantity of interest in Global Sensitivity Analysis as the results can vary depending on the choice of the output parameter. Among the species we see HER4, and HER2 numbers are again significant, along with JAK and cytoplasmic JAK phosphatase SHP2. Here also we see that the total effect sensitivity *S*_*T*_ ≈ *S*_1_ here for all the sensitive parameters which suggests that there is not much higher order effect of parameters on the time-dependent switch.

### Delay in STAT nuclear translocation and β-casein transcription

The next output parameter of interest is the time delay between STAT5 nuclear translocation and β-casein transcription and transport outside the nucleus. Here we make several interesting observations (Fig. 6e and f). The various transport rates in the model especially the rate of activated STAT5 dimer nuclear import and rate of β-casein mRNA nuclear export were prominent in the list of sensitive parameters. The effect of these transport rates on the pathway activity has been reported before in literature [5, 22] and is expected from the model. The SOCS mediated negative feedback rate also features on the sensitive parameters list. The negative feedback does not directly influence the β-casein mRNA levels but has a more indirect effect. The result shows that in a nonlinear system like HER4-JAK-STAT pathway parallel/side reactions can influence the dynamic behavior and can be exploited as therapeutic targets. In contrast to the absolute β-casein sensitivity we see here that *S*_*T*_ ≫ *S*_1_ which suggests that there is a high degree of nonlinear effect of the sensitive parameters to the overall sensitivity of transcription time delay.

In the of the Additional file 1: Figures S1-S2 we explore the effect of high and low NRG stimulation on the global sensitivity coefficients. We see some NRG induced changes in the order of parameters and the degree of nonlinearity, but there were no significant changes in the list of most sensitive parameters.

## Discussion

Epidermal growth factor family of receptors plays a crucial role in different cancers by activating critical signaling pathways that control cell fate decisions. HER4, a member of this family has received attention due to its several distinctive properties. Some isoforms of this receptor can undergo enzyme-mediated cleavage reactions resulting in shedding of its ectodomain and cytoplasmic domain leaving an 80 kDa transmembrane domain which can translocate to the nucleus and promote transcription of various genes. On activation by ligand NRG, it can activate the anti-proliferative JAK2-STAT5A pathway which results in activation, dimerization and nuclear localization of the transcription factor STAT5A which promotes transcription of genes mediating differentiation in particular breast cancer cell lines. This study was motivated by experimental work conducted on mouse HC11 mammary epithelial cell lines stimulated by various ligands including NRG using RT-PCR to measure the expression of the important differentiation marker gene β-casein. One of the intriguing results from the study was that the response of these cells to the NRG stimulation (agonistic versus antagonistic) is time-dependent. It was observed that whereas NRG suppressed the transcription of β-casein at early time points (0-12 h), at later time points (24-48 h), it promoted transcription. Using ELISA based TF activation assays of STAT5A and GR - two transcription factors required for transcription of β-casein, the same study also found that the activity of these transcription factors persisted even 24 h after NRG stimulation. These observations mirrored similar findings in the literature from ChIP-seq studies that showed there is a significant time delay between the STAT5A entry to the nucleus and transcription of the β-casein gene. To obtain a mechanistic understanding of these observations we turned to mathematical modeling. Although the JAK-STAT pathway has been extensively modeled before, the literature models failed to reproduce these experimental findings particularly the time-dependent switch in β-casein gene transcription. The failure of the existing models led us to develop a new model for the HER4-JAK2-STAT5 system that retains the core reactions of the literature models but adds two essential components which have been reported before in the literature but have not been included in the models. These are:

- Competitive binding and heterodimerization of HER4 receptor with other members of the ErbB family like HER3.
- A HER4 mediated JAK-independent activation of STAT5 which proceeds at a lower rate than the canonical JAK-dependent activation.

Including these reactions in our model, we were able to reproduce the experimental findings. By systematically turning these reactions on and off we showed how the signaling dynamics shifted to the pattern seen in the experiments at late time points. We performed extensive parameter sweep studies to understand how different individual components of the model influenced the signaling dynamics. We also conducted global sensitivity analysis to test the robustness of model predictions and to obtain the most sensitive parameters of the model for different output dynamical quantities. These studies show that the competitive HER4 heterodimerization reactions have a profound impact on the sensitivity of the pathway to NRG stimulation at earlier time points. This reaction is a necessary condition for the observed suppression of transcription by NRG.

Along with this competitive heterodimerization, the addition of a slower JAK-independent mechanism of activation of HER4 was sufficient to reproduce the time-dependent switch in the transcription of the β-casein gene observed in the experiments. We also found that the various transport rates in the model such as STAT5 dimer nuclear import and β-casein mRNA export influences the time delays associated with transcription. The Global sensitivity analysis results confirm these model findings by showing that the parameters of the above reactions are most sensitive to the corresponding model output such as delay. This study highlights the effect of competitive and parallel reaction pathways on both short and long-term dynamics of receptor-mediated signaling. Such time-dependent alterations in the signaling behavior of these pathways are highly consequential in cancer and are one of the main ways tumor cells develop resistance to targeted inhibitors. Identification of such time-dependent changes in signaling dynamics can also help in designing optimal treatment strategies with different dose interval and durations [23, 24]. By obtaining a deeper understanding of the dynamics of such pathways, we will be able to design more efficient drug dosing regimens that can target and exploit the differential dynamics. However, because of the uncertainties inherent in these mechanistic models, there may be alternative mechanisms that might explain the observed data. In those cases, a data-driven clustering based approach [25, 26] which has received attention recently can be used to perform system identification and map dynamical behavior the observed data.

## Conclusion

Using a mathematical model of HER4 mediated JAK-STAT pathway that incorporates recently identified reactions of the HER4 receptor, we were able to reproduce the time-dependent switching behavior of the differentiation marker β-casein milk gene observed in HC11 mouse mammary epithelial cells. The model demonstrated the necessity of competitive heterodimerization of HER4 receptor and a slower JAK-independent activation of transcription factor STAT5 to reproduce the time-dependent switch. Through global sensitivity analysis, we also identified the critical parameters such as HER4 competitive heterodimerization rates and rates of transport of STAT5 transcription factor and β-casein mRNA that profoundly influence the dynamical behavior. This study illustrates the importance of parallel reaction pathways and differences in reaction time scales to produce essential changes in the dynamics of cellular pathways. Such models are valuable means of exploring and designing effective treatment strategies in cancers that can exploit the dynamical behavior of the pathways.

## Materials and methods

### Pathway description

The HER4-JAK-STAT system was modeled as a deterministic reaction network using mass action kinetic equations modeled by ordinary differential equations. The system was modeled as a two-compartment system (for cytoplasm and nucleus) with NRG in the extracellular medium. The species were assumed to be in sufficiently large amounts so that the deterministic approximation applies. The model builds on the canonical JAK-STAT pathway model in the literature [5, 6]. In this pathway, the receptor-JAK2 complex is activated by the ligand. This activated receptor complex, in turn, activates cytoplasmic STAT5 which then dimerizes and translocates to the nucleus. The STAT5 dimer in the nucleus initiates transcription of various genes of which the SOCS mRNA which translates to SOCS protein and exerts negative feedback on the circuit by deactivating the activated receptor-JAK2 complex is of interest. The other gene of interest for the current HC11 system is, of course, β-casein, which on transcription is transported outside the nucleus. This cytoplasmic β-casein is reported in the experiments.

To explain the experimental findings which showed that the canonical pathway is inadequate to explain things like a time-dependent switch in β-casein mRNA levels we incorporated two main modifications of the canonical pathway based on recent literature findings

- HER4 can form both homo and heterodimers with other members of the ErbB family on activation by Neuregulin. These reactions compete with the dimerization and activation reactions of HER4-JAK2 complex. Such competition for ligand has been shown to induce an inverse ligand dependence (signaling activity decreases with increasing ligand stimulation) in these pathways which is also observed in the experiments. These additional homodimerization and heterodimerization steps were modeled using three reactions.
- The requirement of JAK2 for tyrosine phosphorylation of STAT5 was only demonstrated in HB-EGF and Prolactin stimulated pathways [3]. There are other shreds of evidence in the literature that suggest STAT5A can directly interact with HER4 s80 when stimulated by HRG/NRG. Hence in the present work, we incorporated this possibility by introducing a JAK2 independent pathway which allows a direct interaction and activation of the s80/4ICD domain of HER4 with STAT5a. The most important effect of adding this pathway is that being JAK independent, this complex will not be negatively regulated by SOCS to the extent when JAK is present [17] which can increase the overall β-casein gene expression in a time-dependent manner. The rate constants of the activation reactions for this JAK2 independent pathway were taken as smaller than that of the JAK2-dependent pathway since we expect the active kinase domain of JAK2 to catalyze the phosphorylation of STAT5.

The rate constants were taken from published models [5, 6, 27] for the reactions which were common to the current model. For the new reactions, we estimated the rate constants by starting with similar values as the related reactions for which the parameters are known, and then doing sensitivity analysis and comparing with experimental results. Bounds for the rate constants were also confirmed independently using values/estimates of the diffusion coefficient. The model was solved using COPASI [28]. A full description of the model along with the initial expressions and rate constants are provided in the SBML (http://sbml.org/Documents/Specifications) format in Additional file 2. We used Latin Hypercube sampling [29] to sample the parameter space and to create an ensemble of models. Then for each member of the ensemble, we calculate and plot the time-integrated value of β-casein mRNA and average them over the ensemble. This task ensures that the uncertainties in the model parameters are reflected in the predictions to a certain degree and that the model is robust to small perturbations in the model parameters.

### Global sensitivity analysis

Real biological pathways like HER4-JAK2-STAT5 are nonlinear and various parameters such as initial species expression, kinetic rate constants, etc. can undergo large deviations. Sensitivity analysis techniques are useful to understand how perturbations in these parameters influence the model outputs. Because these systems have a high degree of nonlinearity, simple local sensitivity analysis where each parameter is altered one at a time keeping the others fixed is not sufficient [20]. Hence, we use a global sensitivity analysis for this model – more specifically the Sobol Sensitivity Analysis which is based on analysis of variances in the model parameters and output.

### Sobol sensitivity analysis

Sobol sensitivity analysis is a variance-based technique and is uniquely well suited for complex nonlinear systems of moderate size. Here we give a very brief description of the method that is relevant to the sensitivity analysis done in this paper. A detailed description can be found in references [21, 30]. We use many of the notations from [30] below.

Suppose we have a model with k parameters *X*_1_, *X*_2_, … , *X*_*k*_ which are assumed to be independent random variables. The model output Y is related to these parameters through the relation *Y* = *f*(*X*_1_, *X*_2_,  … , *X*_*k*_) where f is a general nonlinear function. In a variance-based sensitivity analysis, we want to understand how variances in the individual and combination of parameters *X*_1_, *X*_2_, … , *X*_*k*_ factor into the variances in Y. To determine this, we can first fix a parameter *X*_*i*_ to a value (say *V*_*i*_) and then determine the model output averaged over all remaining parameters *X*_*j*_, *i* ≠ *j* (which is denoted with a condensed notation ${\overline{X}}_{\sim i}$). This average is ${E}_{{\overline{X}}_{\sim i}}\left(Y|{X}_i={V}_i\right)$ which will be different for different *V*_*j*_. The variance of this average over all possible *V*_*i*_ which is ${V}_{X_i}\left({E}_{{\overline{X}}_{\sim i}}\left(Y|{X}_i={V}_i\right)\right)$ will give us the net first order effect of variation in *X*_*i*_ on the variation in *Y*. The first order sensitivity *S*_*i*_ associated with parameter *X*_*i*_ is defined as:

$$ {S}_i=\frac{V_{X_i}\left({E}_{{\overline{X}}_{\sim i}}\left(Y|{X}_i={V}_i\right)\right)}{V(Y)} $$

In the above *V*(*Y*) is the overall variance in Y.

The other sensitivity parameter of interest is the total effect sensitivity ${S}_{T_i}$ which represents the first and all higher order effects of the parameter *X*_*i*_ on the model output. To determine this, we can start with determining the first order effect of all parameters except *X*_*i*_ which is denoted by ${\overline{X}}_{\sim i}$. Again, we first find the value of Y averaged over all *X*_*i*_ keeping all other parameters ${\overline{X}}_{\sim i}$ fixed which is ${E}_{X_i}\left(Y|{\overline{X}}_{\sim i}={\overline{V}}_{\sim i}\right)$. We then find the variance of this quantity over all possible ${\overline{X}}_{\sim i}$ or ${V}_{{\overline{X}}_{\sim i}}\left(\ {E}_{X_i}\left(Y|{\overline{X}}_{\sim i}={\overline{V}}_{\sim i}\right)\right)$. Then $V(Y)-{V}_{{\overline{X}}_{\sim i}}\left(\ {E}_{X_i}\left(Y|{\overline{X}}_{\sim i}={\overline{V}}_{\sim i}\right)\right)$ must represent the contribution of all terms where *X*_*i*_ appears. Dividing this by *V*(*Y*) we get the total effect sensitivity:

$$ {S}_{T_i}=\frac{V(Y)-{V}_{{\overline{X}}_{\sim i}}\left(\ {E}_{X_i}\left(Y|{\overline{X}}_{\sim i}={\overline{V}}_{\sim i}\right)\right)}{V(Y)}=1-\frac{V_{{\overline{X}}_{\sim i}}\left(\ {E}_{X_i}\left(Y|{\overline{X}}_{\sim i}={\overline{V}}_{\sim i}\right)\right)}{V(Y)} $$

This variance-based sensitivity analysis framework is based on a functional decomposition scheme where a square integrable function *Y* = *f*(*X*_1_, *X*_2_,  … , *X*_*k*_) defined over Ω, the k-dimensional unit hypercube, can be expressed as follows:

$$ f={f}_0+\sum \limits_i{f}_i+\sum \limits_i\sum \limits_{j>i}{f}_{ij}+\dots +{f}_{12\dots k} $$

The normalization condition here is

$$ {\int}_0^1{f}_{i_1\dots {i}_s}\left({x}_{i_1},{x}_{i_2},\dots, {x}_{i_s}\right)d{x}_w=0 $$

In the above *w* = *i*_1_, … , *i*_*s*_. Using this the various terms are calculated as

$$ {f}_0=\int f(x)\  dx=E(Y) $$

$$ {f}_i\left({x}_i\right)=\int f(x)\prod \limits_{w\ne i}d{x}_w-{f}_0={E}_{{\overline{X}}_{\sim i}}\left(Y|{X}_i\right)-E(Y) $$

$$ {f}_{ij}\left({x}_i,{x}_j\right)=\int f(x)\prod \limits_{w\ne i,j}d{x}_w-{f}_0-{f}_i\left({x}_i\right)-{f}_j\left({x}_j\right)={E}_{{\overline{X}}_{\sim ij}}\left(Y|{X}_i,{X}_j\right)-{E}_{{\overline{X}}_{\sim i}}\left(Y|{X}_i\right)-{E}_{{\overline{X}}_{\sim j}}\left(Y|{X}_j\right)-E(Y) $$

Taking the variances of both sides of these equations give us

$$ {V}_i=V\left({f}_i\right)={V}_{X_i}\left({E}_{{\overline{X}}_{\sim i}}\left(Y|{X}_i\right)\right) $$

$$ {V}_{ij}=V\left({f}_{ij}\right)={V}_{X_i{X}_j}\left({E}_{{\overline{X}}_{\sim ij}}\left(Y|{X}_i,{X}_j\right)\right)-{V}_{X_i}\left({E}_{{\overline{X}}_{\sim i}}\left(Y|{X}_i\right)\right)-{V}_{X_j}\left({E}_{{\overline{X}}_{\sim j}}\left(Y|{X}_j\right)\right) $$

All these variances are linked by

$$ V(Y)=\sum \limits_i{V}_i+\sum \limits_i\sum \limits_{j>i}{V}_{ij}+\dots +{V}_{12\dots k} $$

Dividing the above by *V*(*Y*), we get

$$ \sum \limits_i{S}_i+\sum \limits_i\sum \limits_{j>i}{S}_{ij}+\dots +{S}_{12\dots k}=1 $$

Since the sensitivity coefficients above are multidimensional integrals, the standard way of computing them is by using Monte Carlo type sampling. In Monte Carlo sampling an integral *I*[*f*] =  ∫ *f*(*x*)*dx* is computed by generating a sequence of uniformly distributed random numbers and computing their expectation ${I}_N\left[f\right]=\frac{1}{N}{\sum}_{i=1}^Nf\left({x}_i\right)$. In this case, we have a k-dimensional function *Y* = *f*(*X*_1_, *X*_2_,  … , *X*_*k*_). Hence, we need to sample N times for each of these k parameters. These samples can be represented by a *N* × *k* matrix. For the calculation of the above sensitivities, the standard procedure is to start with two independent *N* × *k* sampling matrices $\overline{\overline{A}}$ and $\overline{\overline{B}}$. We can compute matrices ${\overline{\overline{A}}}_B^i$ which is obtained by taking $\overline{\overline{A}}$ and replacing the ith column (for parameter *X*_*i*_) by the corresponding column from $\overline{\overline{B}}$. Similarly, we can define ${\overline{\overline{B}}}_A^i$. It can be shown that [30] the variances in the equations for *S*_*i*_ and ${S}_{T_i}$ can be estimated using

$$ {V}_{X_i}\left({E}_{{\overline{X}}_{\sim i}}\left(Y|{X}_i={V}_i\right)\right)=\frac{1}{N}\sum \limits_{j=1}^Nf{\left(\overline{\overline{A}}\right)}_jf{\left({\overline{\overline{B}}}_A^i\right)}_j-{f}_0^2 $$

$$ {V}_{{\overline{X}}_{\sim i}}\left(\ {E}_{X_i}\left(Y|{\overline{X}}_{\sim i}={\overline{V}}_{\sim i}\right)\right)=\frac{1}{N}\sum \limits_{j=1}^Nf{\left(\overline{\overline{A}}\right)}_jf{\left({\overline{\overline{A}}}_B^i\right)}_j-{f}_0^2 $$

The above equations form the basis of the computation of the sensitivity coefficients for the model.

### Quasi-random sequences

Monte Carlo method using a sequence of random or pseudorandom numbers is extensively used to compute multidimensional integrals like above. The Central Limit Theorem of probability shows that the error in the Monte Carlo estimate is equal to the product of the standard deviation of the function and ${N}^{-\frac{1}{2}}$ where N is the number of samples. Hence the convergence of this method is $O\left({N}^{-\frac{1}{2}}\right)$ which can be very slow [31]. This method of sampling using pseudorandom numbers also suffers from a related problem of clumping where the sample points often tend to clump together and leaves empty spaces in between which is magnified in higher dimensions. One alternative to obtaining a more uniform distribution of points is by using a stratified sampling method like Latin Hypercube Sampling which divides the intervals into equally spaced points. However, this only works when the dimensionality is low. For integrations in higher dimensions, an alternative sampling technique is applied called quasi-random sampling. A quantitative measure of uniformity of a sequence is a factor termed “*discrepancy”*. Lower the discrepancy, more uniform is the sequence. Suppose we have a sequence of N points {*x*_*N*_} in the k dimensional unit cube *I*^*k*^. For any subset *J* ∈ *I*^*k*^ one can define the error in Monte Carlo estimate of the volume of *J* as [31]:

$$ {R}_N(J)=\frac{1}{N}\#\left\{{x}_N\in J\right\}-m(J) $$

The discrepancy is then defined as some norm of *R*_*N*_(*J*). Formally, if J is restricted to rectangular set and E is all possible such sets then the discrepancy *D*_*N*_ is defined as

$$ {D}_N=\underset{J\in E}{\sup}\left|{R}_N(J)\right| $$

The Koksma-Hlawka inequality provides an upper bound for a Monte Carlo integration error as a product of the variance of the function and the discrepancy of the sequence. Hence, if one can generate a sequence of points in such a way as to minimize the discrepancy, then it can be used to obtain an improved estimate of the integral. Such sequences are called quasi-random sequences. These are not random at all but are generated deterministically to minimize discrepancy. Since they are not random, quasi-random sequences are more limited in scope than pseudorandom numbers. However, it can be shown that for integration the convergence rate of quasi-random sequences is *O*(*N*^−1^(log*N*)^*k*^) where k is any number which is considerably faster than the $O\left({N}^{-\frac{1}{2}}\right)$ convergence of the standard Monte-Carlo method using pseudorandom sequences. There are various techniques for determining quasi random sequences. We use Sobol sequences [32] using a method suggested by Saltelli [30]. The software package SALib [33] was used for the computation of the Sobol coefficients along with custom python scripts and matplotlib [34] for plotting.

## Additional files

Additional file 1: Collection of all the supplementary figures showing the results of sensitivity analysis and parameter sweep studies (PDF 2789 kb)

Additional file 2: The detailed HER4-JAK-STAT model with the reactions, initial expression of the proteins and the reaction rate constants in Systems Biology Markup Language (SBML) format. (XML 58 kb)
