# Supplementary material for: Time-dependent antagonist-agonist switching in receptor tyrosine kinase-mediated signaling
Source: BMC Bioinformatics. 2019 May 15;20:242. doi: 10.1186/s12859-019-2816-3 (PMC6521356; doi:10.1186/s12859-019-2816-3)
Supplement: Supplementary file 1 — Collection of all the supplementary figures showing the results of sensitivity analysis and parameter sweep studies (PDF 2789 kb) [file 12859_2019_2816_MOESM1_ESM.pdf]

Supplementary Figures

Figure 1

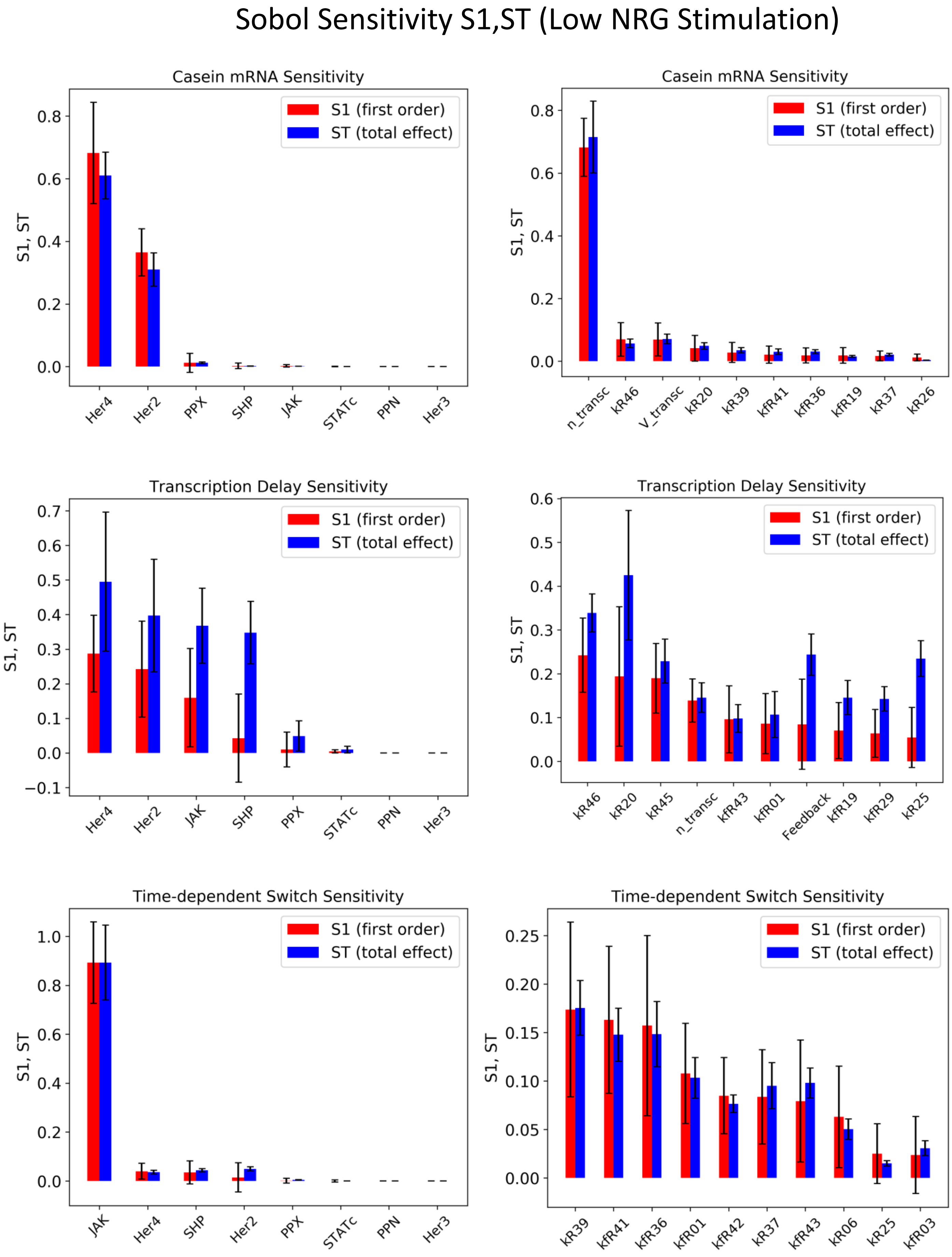
Sobol Sensitivity S1,ST (Low NRG Stimulation)
Casein mRNA Sensitivity
S1 (first order)
ST (total effect)
S1, ST
Her4
Her2
PPX
SHP
JAK
STATc
PPN
Her3
Casein mRNA Sensitivity
n_transc
kR46
V_transc
kR20
kR39
kfR41
kfR36
kfR19
kR37
kR26
Transcription Delay Sensitivity
Her4
Her2
JAK
SHP
PPX
STATc
PPN
Her3
Transcription Delay Sensitivity
kR46
kR20
kR45
n_transc
kfR43
kfR01
Feedback
kfR19
kfR29
kR25
Time-dependent Switch Sensitivity
JAK
Her4
SHP
Her2
PPX
STATc
PPN
Her3
Time-dependent Switch Sensitivity
kR39
kfR41
kfR36
kfR01
kfR42
kR37
kfR43
kR06
kR25
kfR03

Figure 2

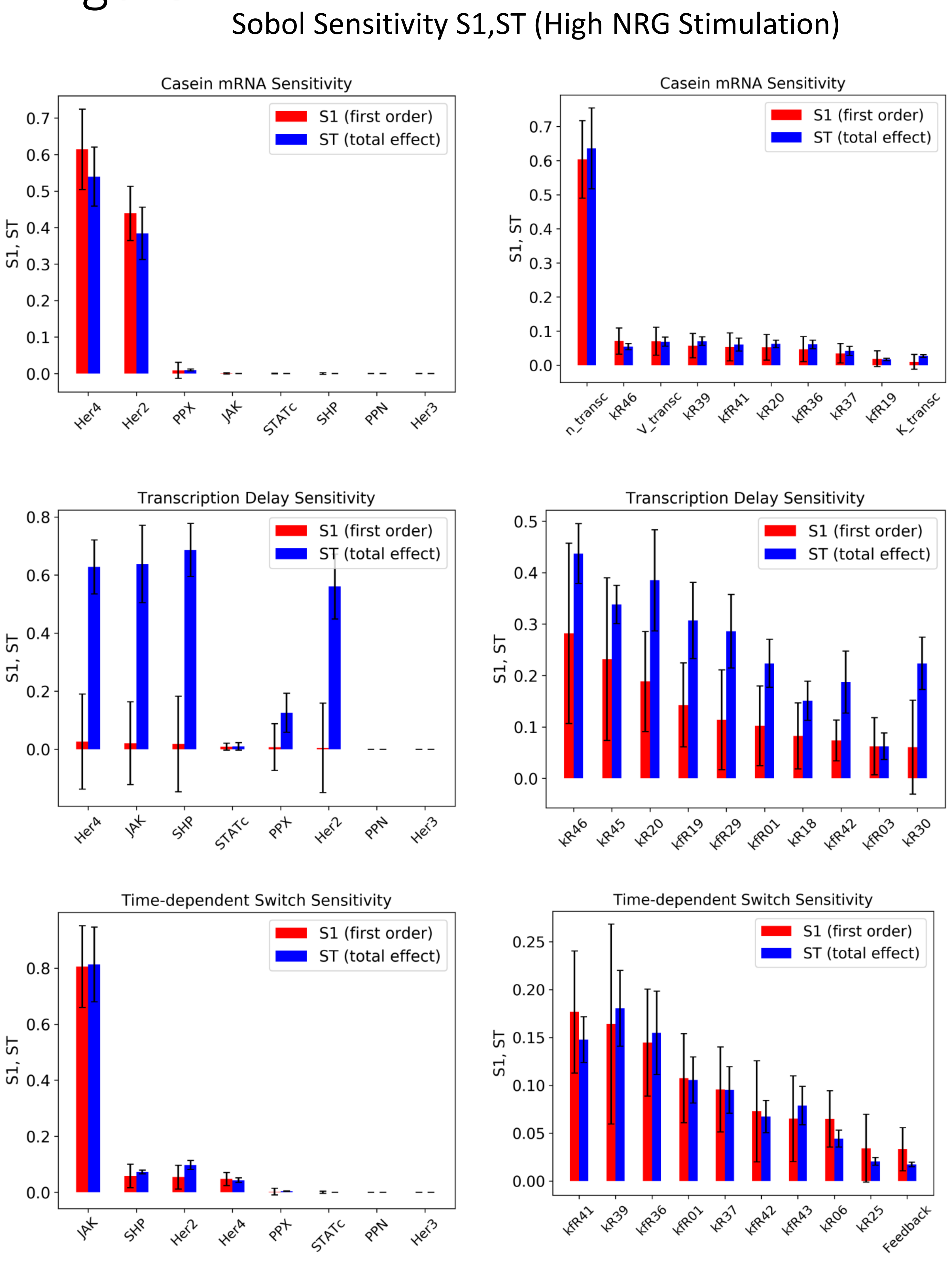

Sobol Sensitivity S1,ST (High NRG Stimulation)
Casein mRNA Sensitivity
S1 (first order)
ST (total effect)
S1, ST
Her4
Her2
PPX
JAK
STATc
SHP
PPN
Her3
Casein mRNA Sensitivity
n_transc
kR46
V_transc
kR39
kfR41
kR20
kfR36
kR37
kfR19
K_transc
Transcription Delay Sensitivity
Her4
JAK
SHP
STATc
PPX
Her2
PPN
Her3
Transcription Delay Sensitivity
kR46
kR45
kR20
kfR19
kfR29
kfR01
kR18
kfR42
kfR03
kR30
Time-dependent Switch Sensitivity
JAK
SHP
Her2
Her4
PPX
STATc
PPN
Her3
Time-dependent Switch Sensitivity
kfR41
kR39
kfR36
kfR01
kR37
kfR42
kfR43
kR06
kR25
Feedback

Figure 5

Initial HER2 (Low)

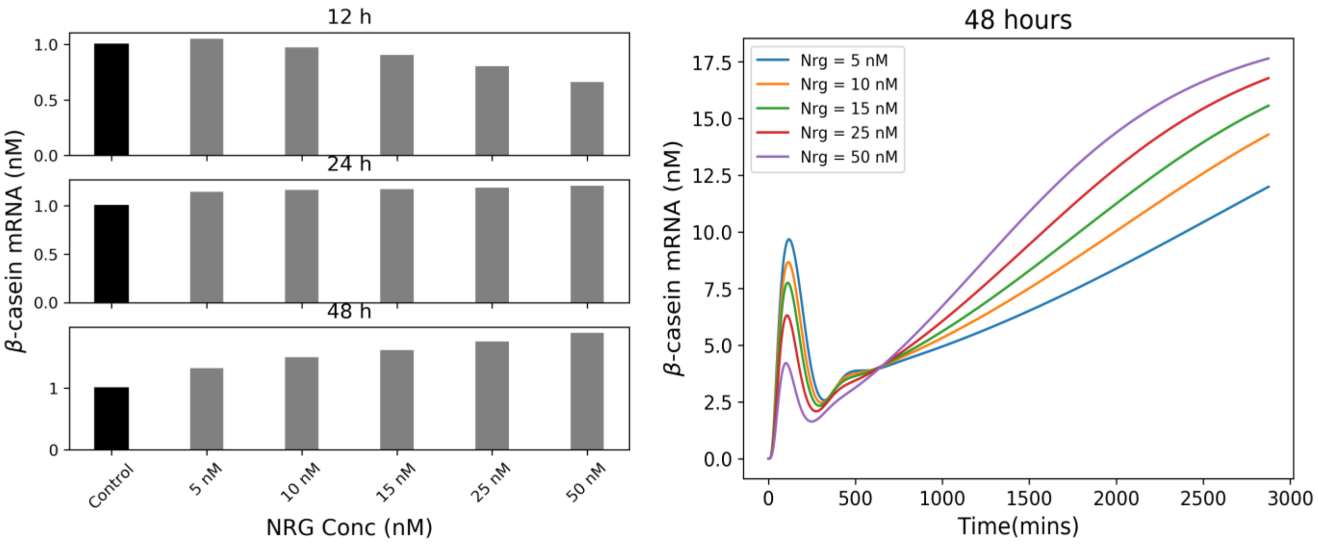

Initial HER2 (Medium/Base)

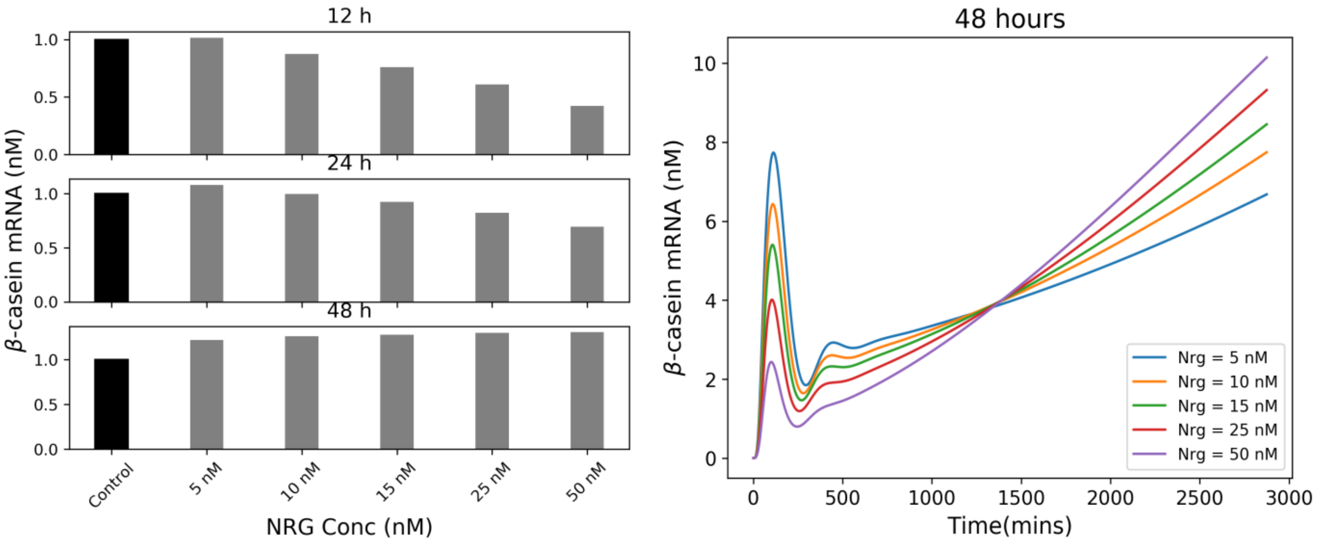

Initial HER2 (High)

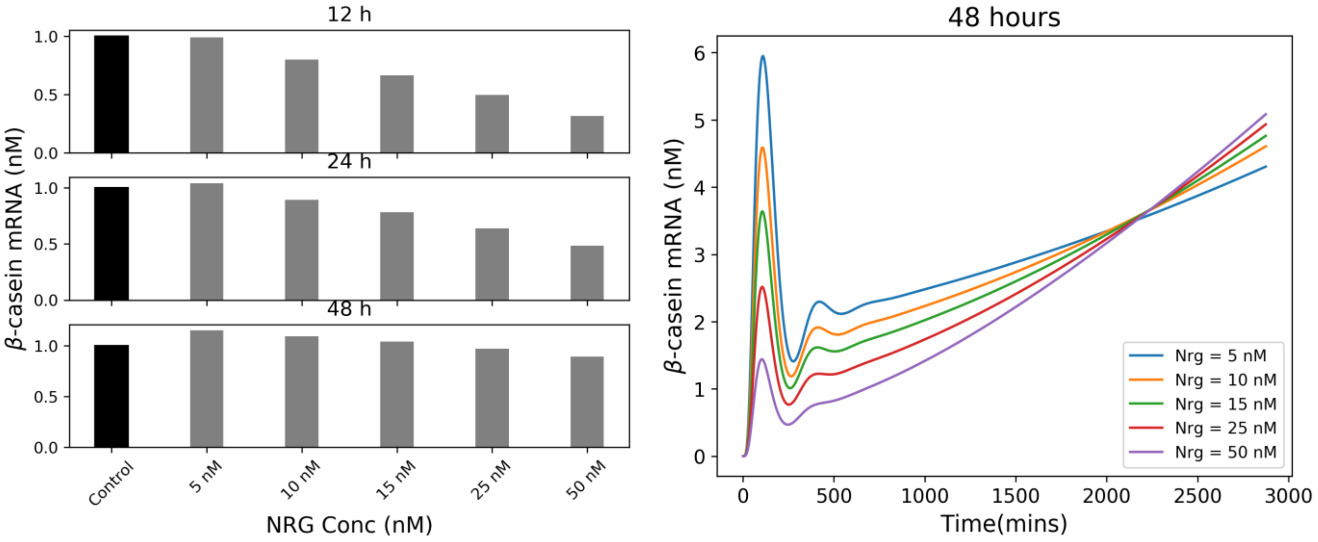

Figure 6

Initial HER4 (Low)

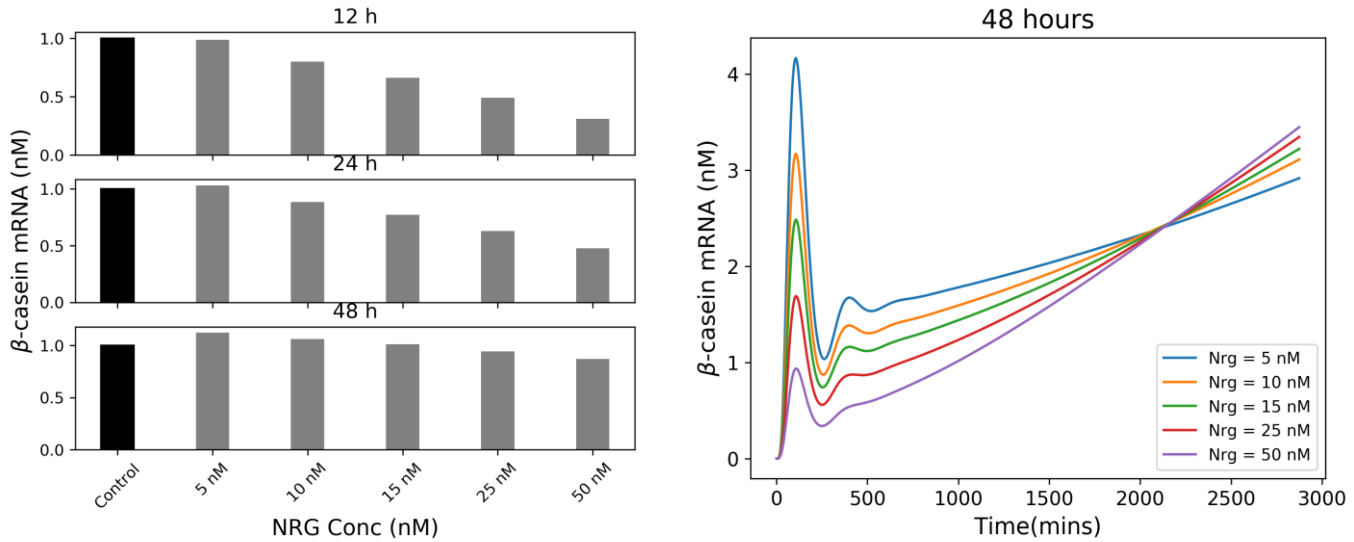

Initial HER4 (Medium/Base)

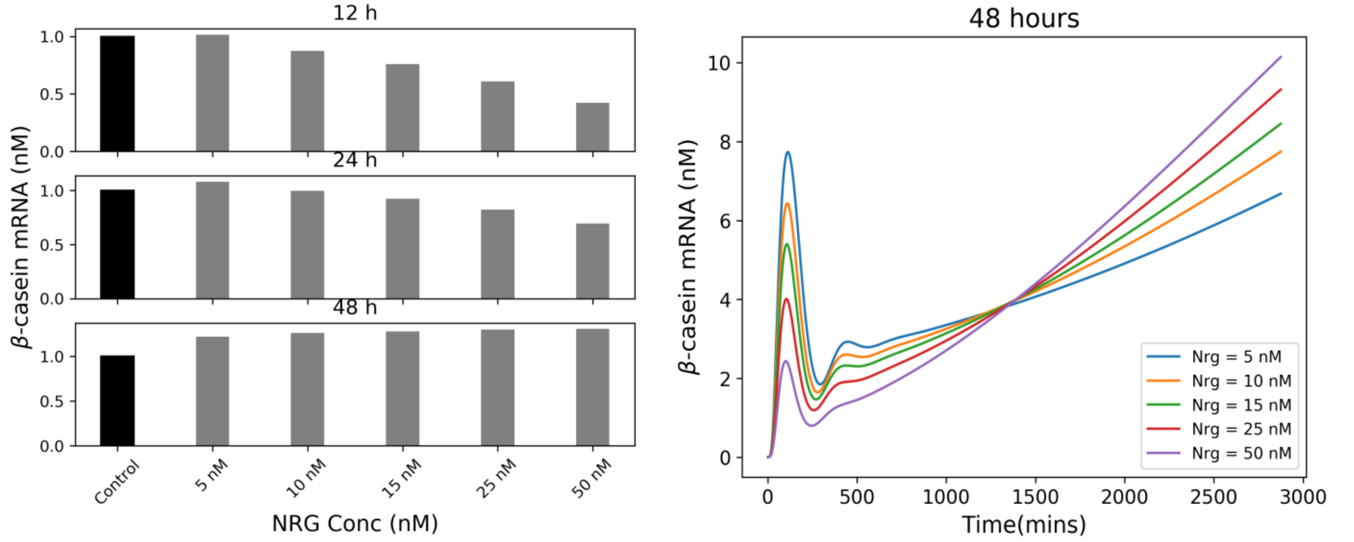

Initial HER4 (High)

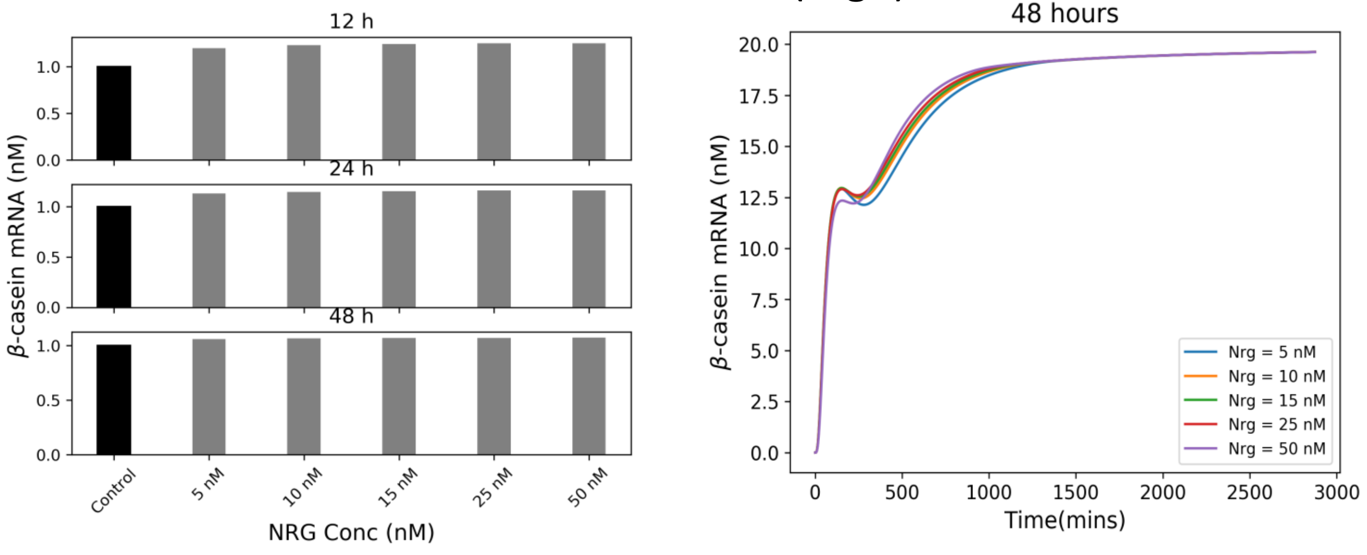

Figure 7

Initial JAK (Low)

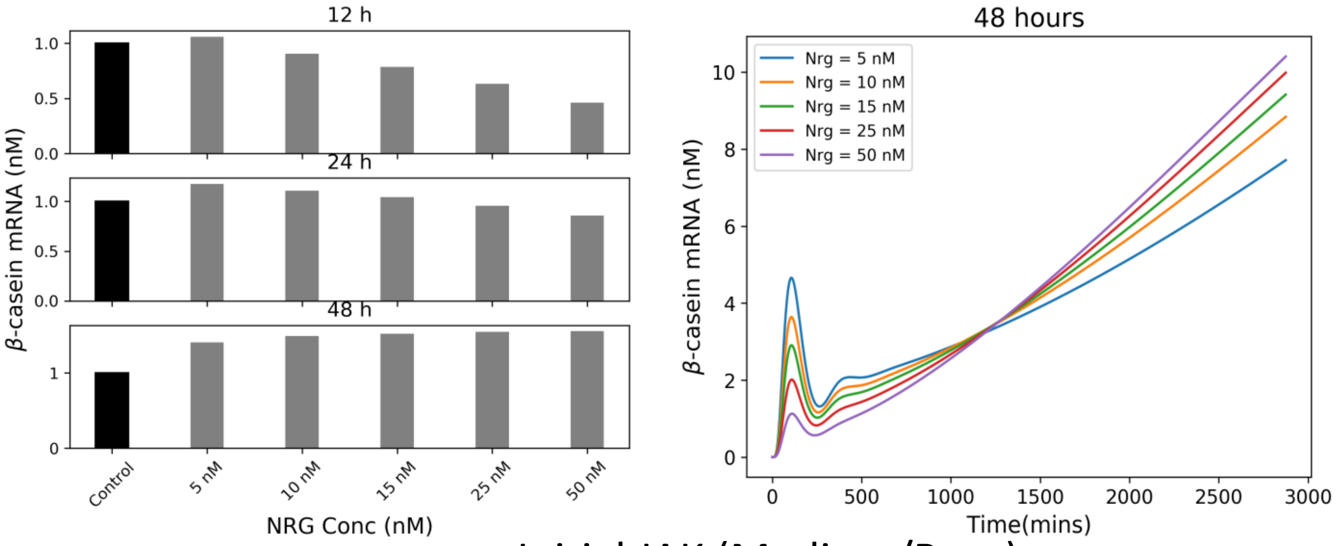

Initial JAK (Medium/Base)

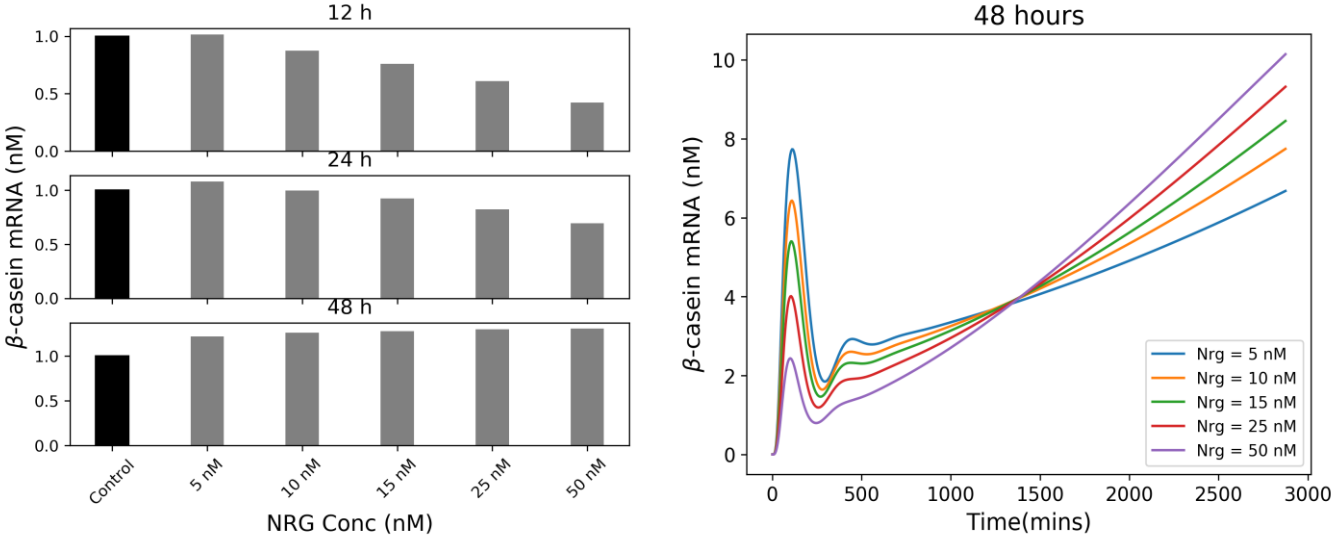

Initial JAK (High)

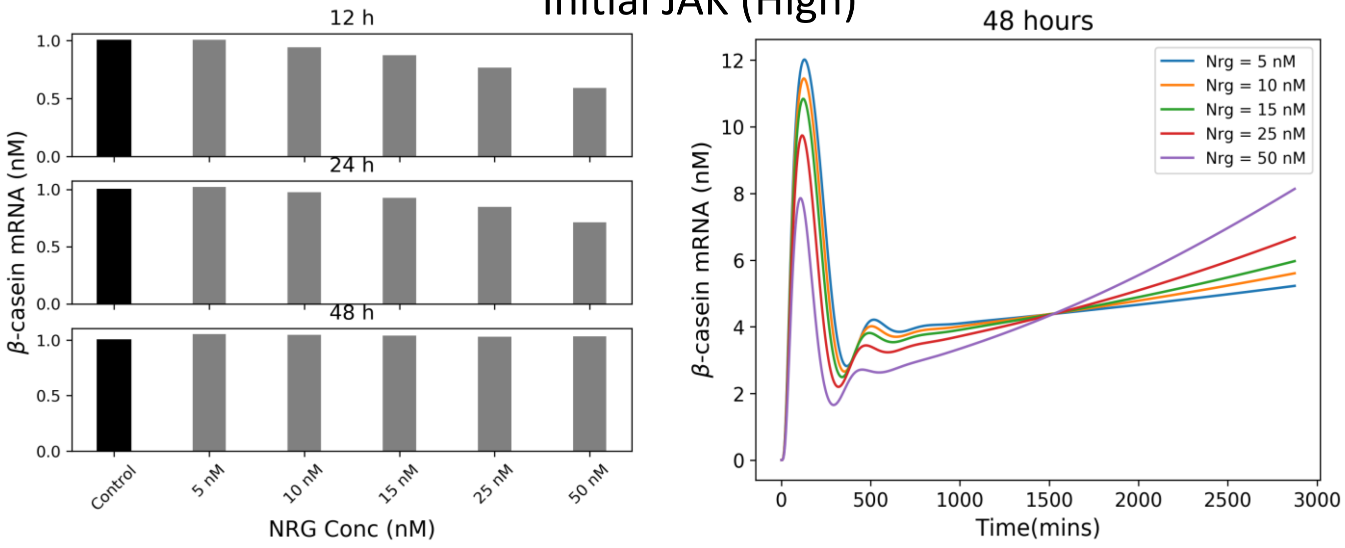

Figure 8

Initial PPN (Low)

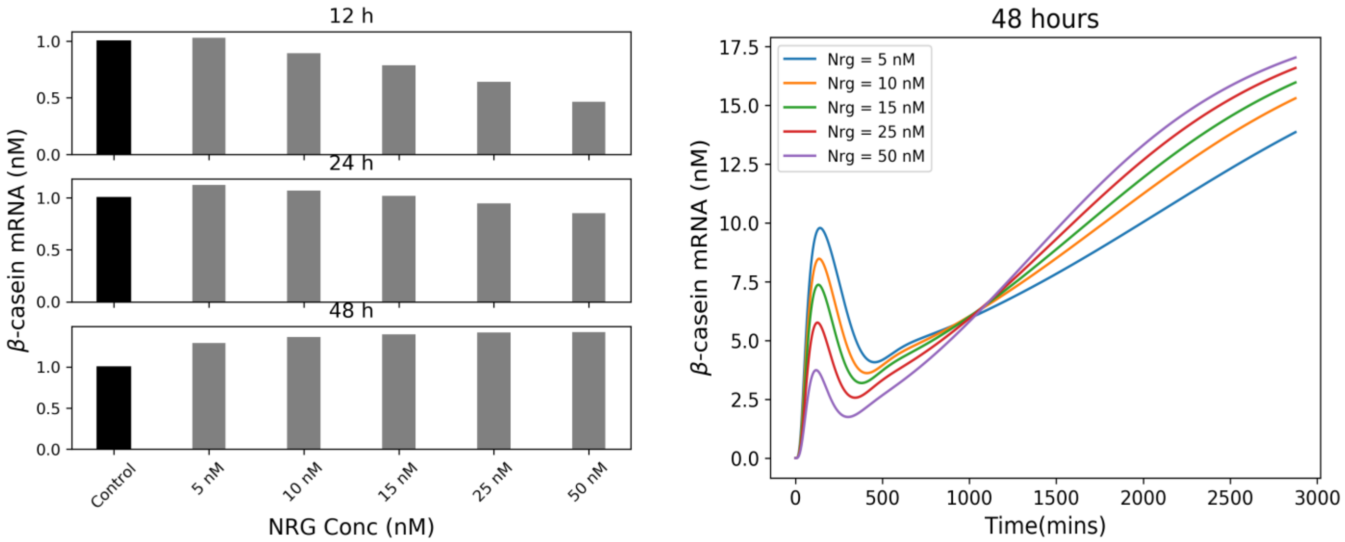

Initial PPN (Medium/Base)

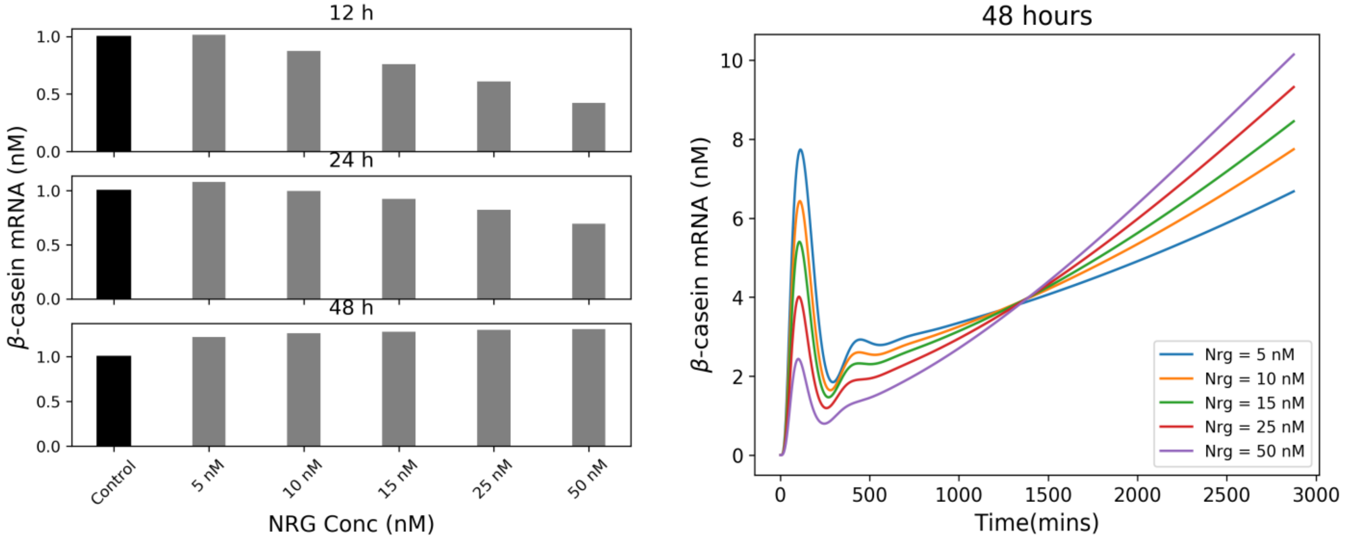

Initial PPN (High)

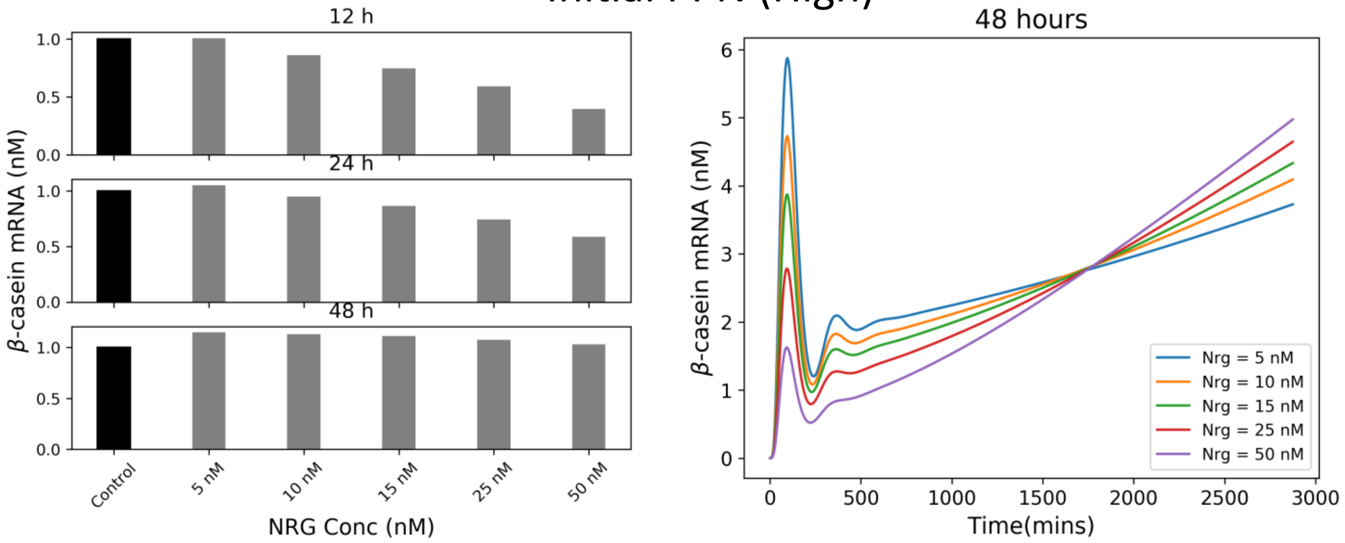

Figure 9

Initial PPX (Low)

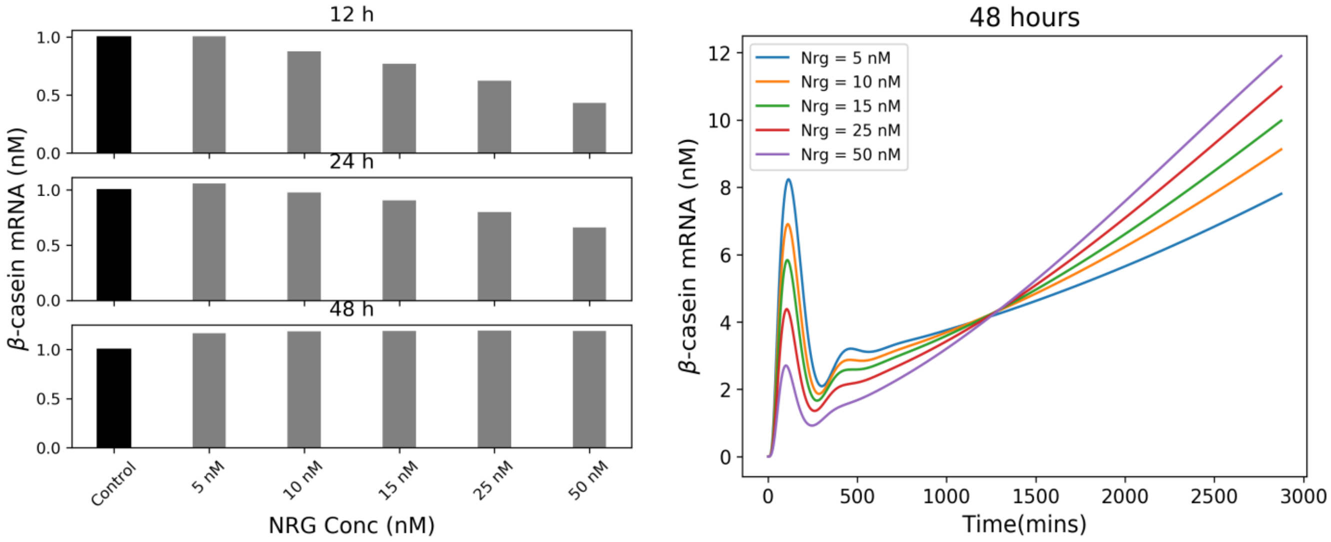

Initial PPX (Medium/Base)

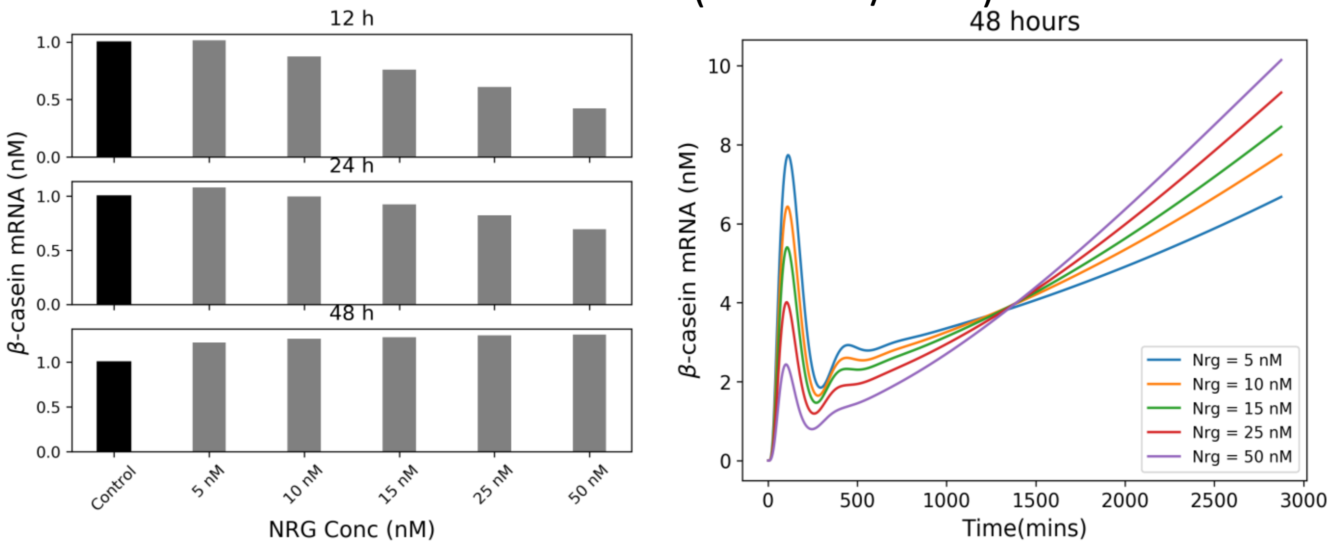

Initial PPX (High)

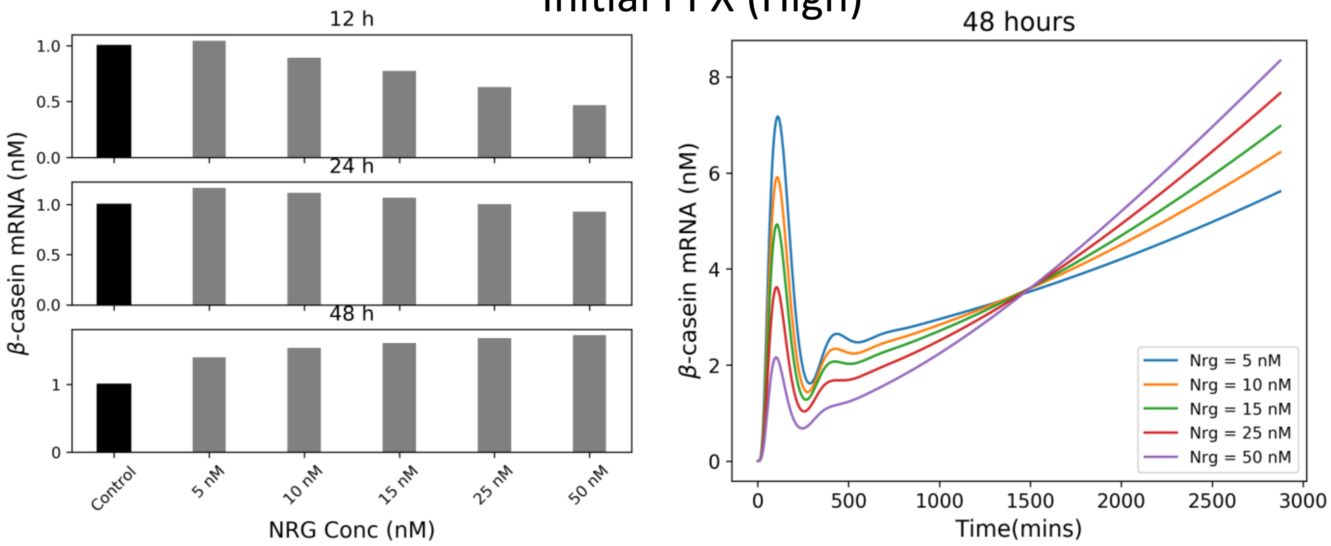

Figure 10

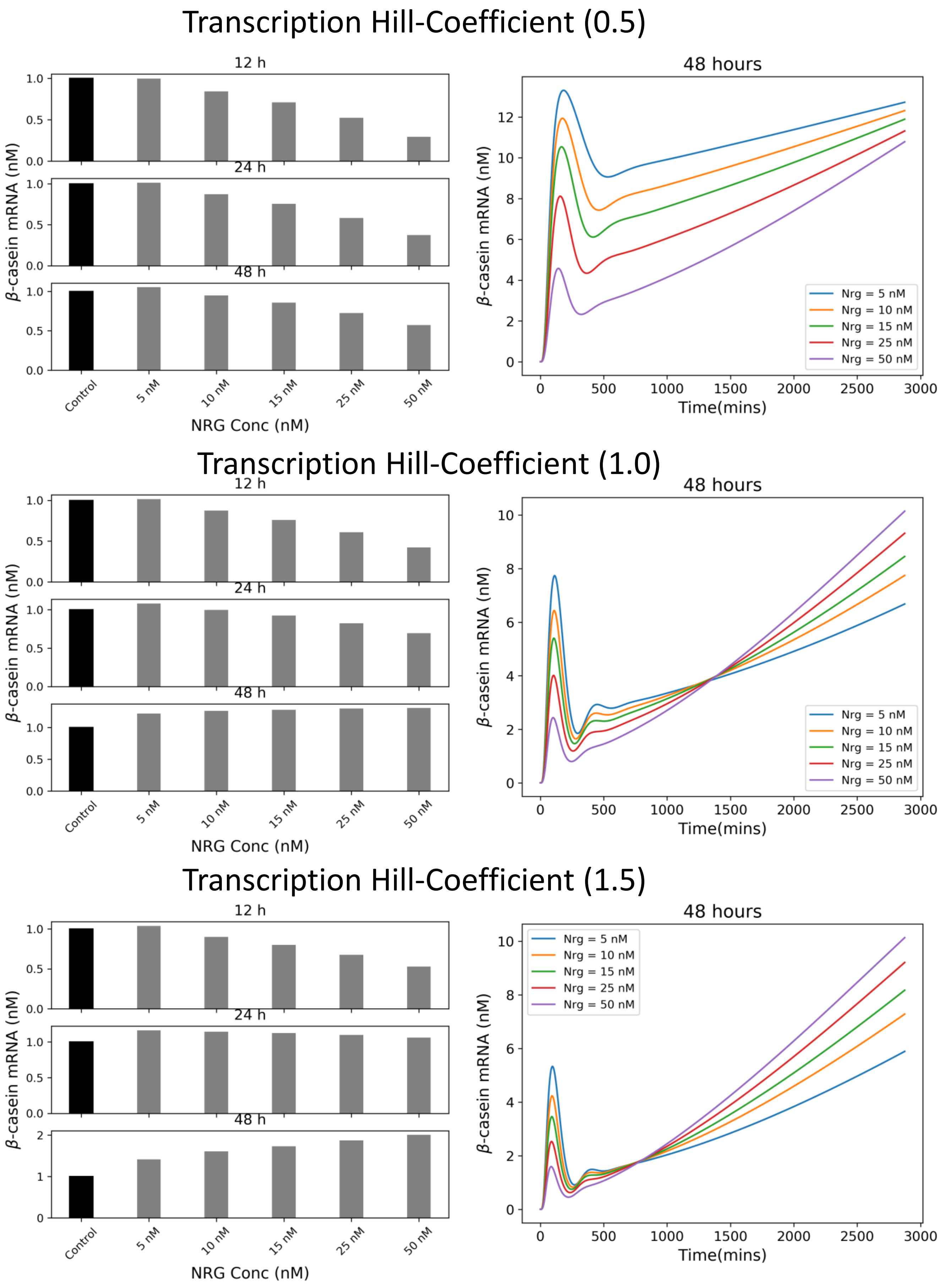

Transcription Hill-Coefficient (0.5)
12 h
24 h
48 h
β-casein mRNA (nM)
1.0
0.5
0.0
Control
5 nM
10 nM
15 nM
25 nM
50 nM
NRG Conc (nM)
48 hours
β-casein mRNA (nM)
0
2
4
6
8
10
12
0
500
1000
1500
2000
2500
3000
Time(mins)
Nrg = 5 nM
Nrg = 10 nM
Nrg = 15 nM
Nrg = 25 nM
Nrg = 50 nM
Transcription Hill-Coefficient (1.0)
12 h
24 h
48 h
β-casein mRNA (nM)
1.0
0.5
0.0
Control
5 nM
10 nM
15 nM
25 nM
50 nM
NRG Conc (nM)
48 hours
β-casein mRNA (nM)
0
2
4
6
8
10
0
500
1000
1500
2000
2500
3000
Time(mins)
Nrg = 5 nM
Nrg = 10 nM
Nrg = 15 nM
Nrg = 25 nM
Nrg = 50 nM
Transcription Hill-Coefficient (1.5)
12 h
24 h
48 h
β-casein mRNA (nM)
1.0
0.5
0.0
2
1
0
Control
5 nM
10 nM
15 nM
25 nM
50 nM
NRG Conc (nM)
48 hours
β-casein mRNA (nM)
0
2
4
6
8
10
0
500
1000
1500
2000
2500
3000
Time(mins)
Nrg = 5 nM
Nrg = 10 nM
Nrg = 15 nM
Nrg = 25 nM
Nrg = 50 nM

Figure 11

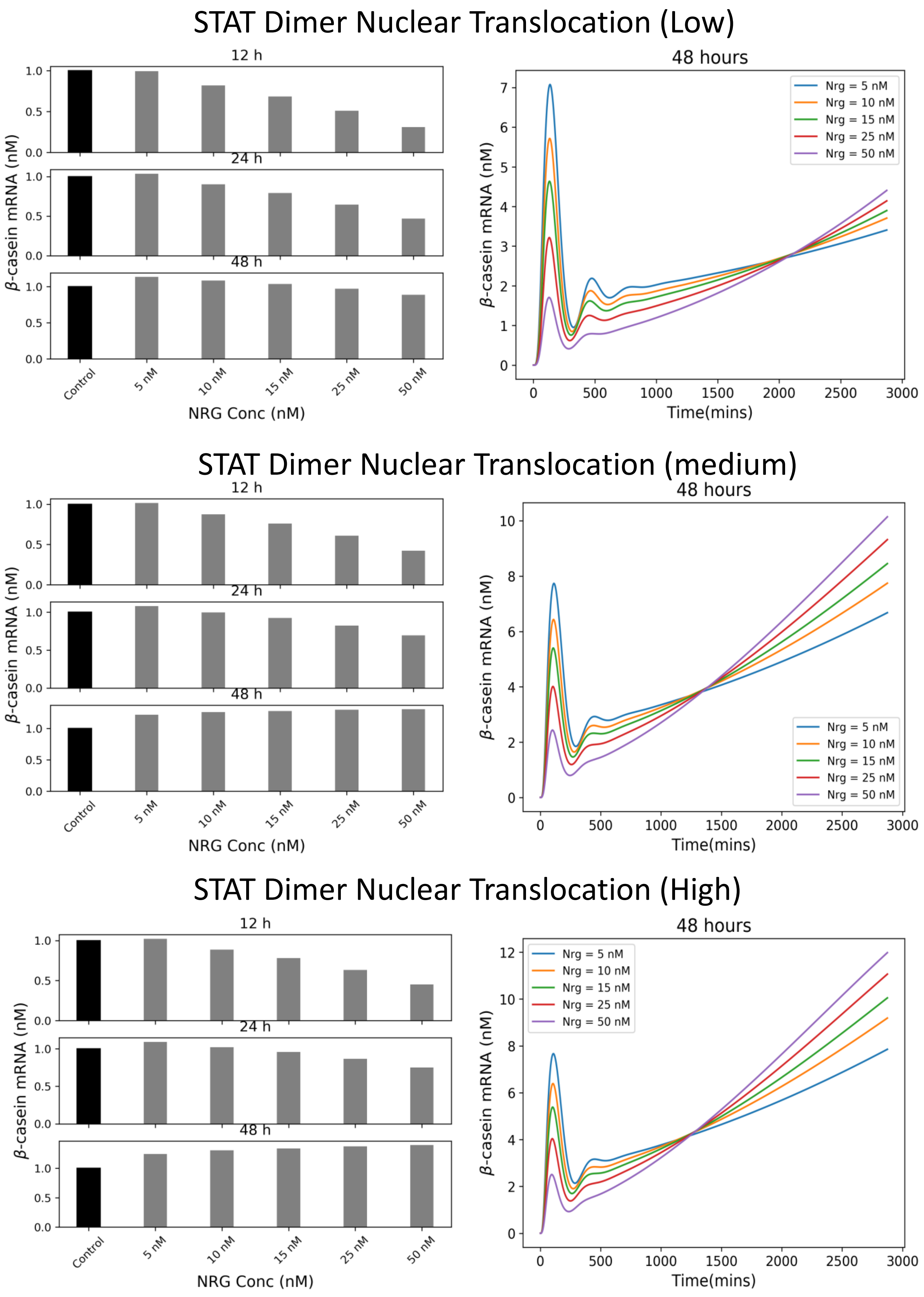
STAT Dimer Nuclear Translocation (Low)
12 h
24 h
48 h
β-casein mRNA (nM)
Control
5 nM
10 nM
15 nM
25 nM
50 nM
NRG Conc (nM)
48 hours
Nrg = 5 nM
Nrg = 10 nM
Nrg = 15 nM
Nrg = 25 nM
Nrg = 50 nM
β-casein mRNA (nM)
Time(mins)
STAT Dimer Nuclear Translocation (medium)
12 h
24 h
48 h
β-casein mRNA (nM)
Control
5 nM
10 nM
15 nM
25 nM
50 nM
NRG Conc (nM)
48 hours
Nrg = 5 nM
Nrg = 10 nM
Nrg = 15 nM
Nrg = 25 nM
Nrg = 50 nM
β-casein mRNA (nM)
Time(mins)
STAT Dimer Nuclear Translocation (High)
12 h
24 h
48 h
β-casein mRNA (nM)
Control
5 nM
10 nM
15 nM
25 nM
50 nM
NRG Conc (nM)
48 hours
Nrg = 5 nM
Nrg = 10 nM
Nrg = 15 nM
Nrg = 25 nM
Nrg = 50 nM
β-casein mRNA (nM)
Time(mins)

Figure 12

HER4 Constitutive Homodimerization (Low)

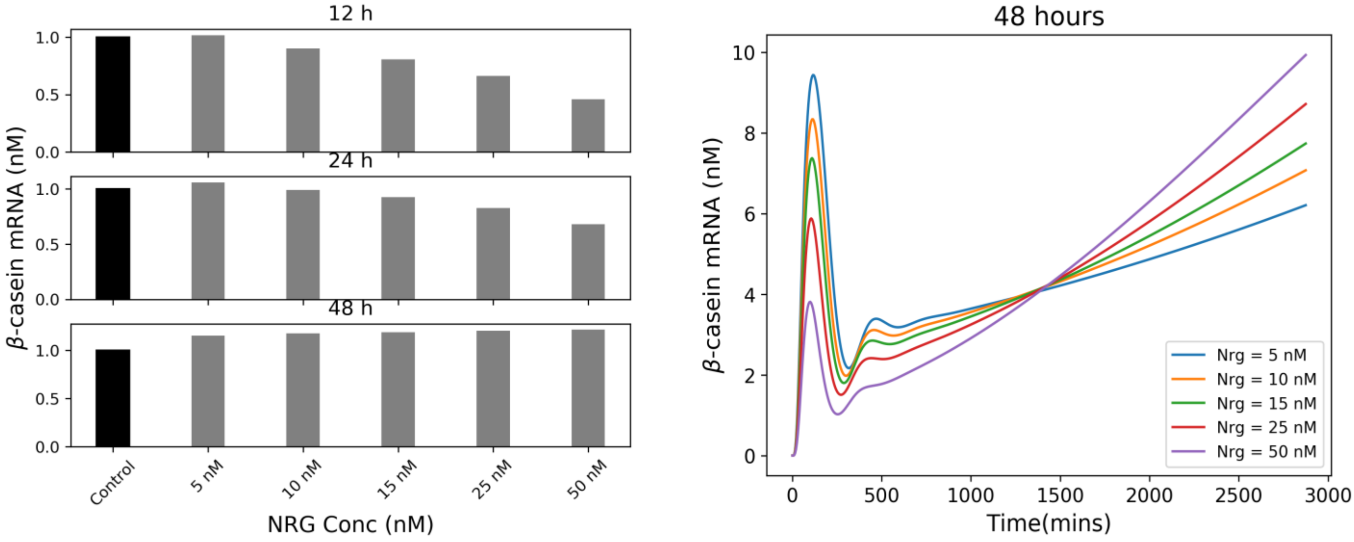

HER4 Constitutive Homodimerization (Medium/Baseline)

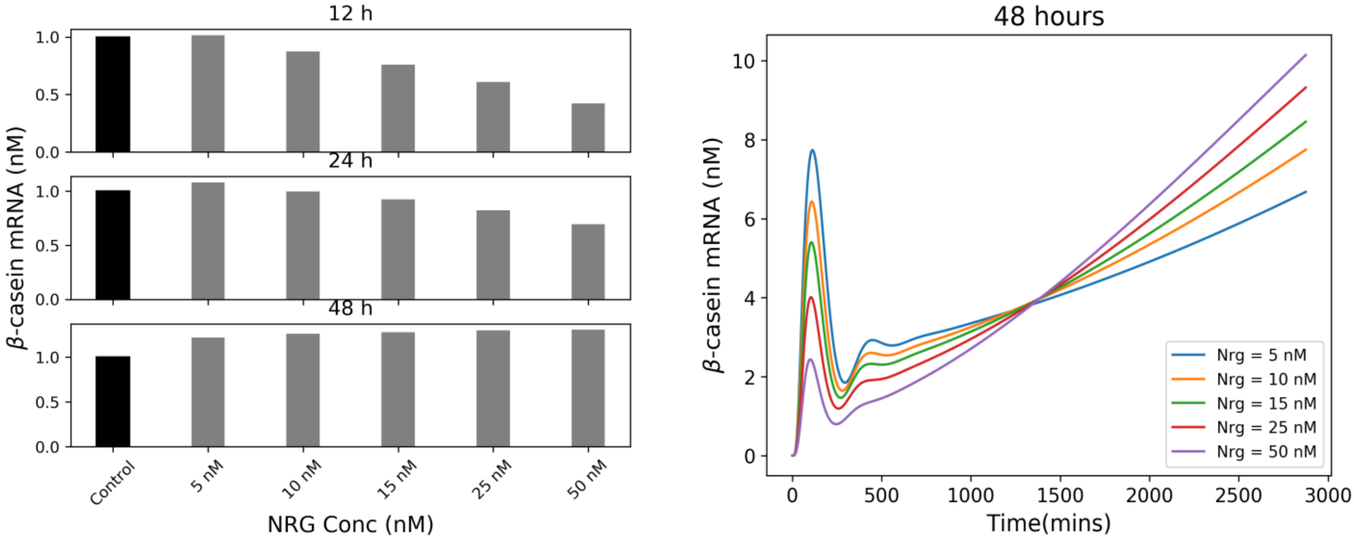

HER4 Constitutive Homodimerization (High)

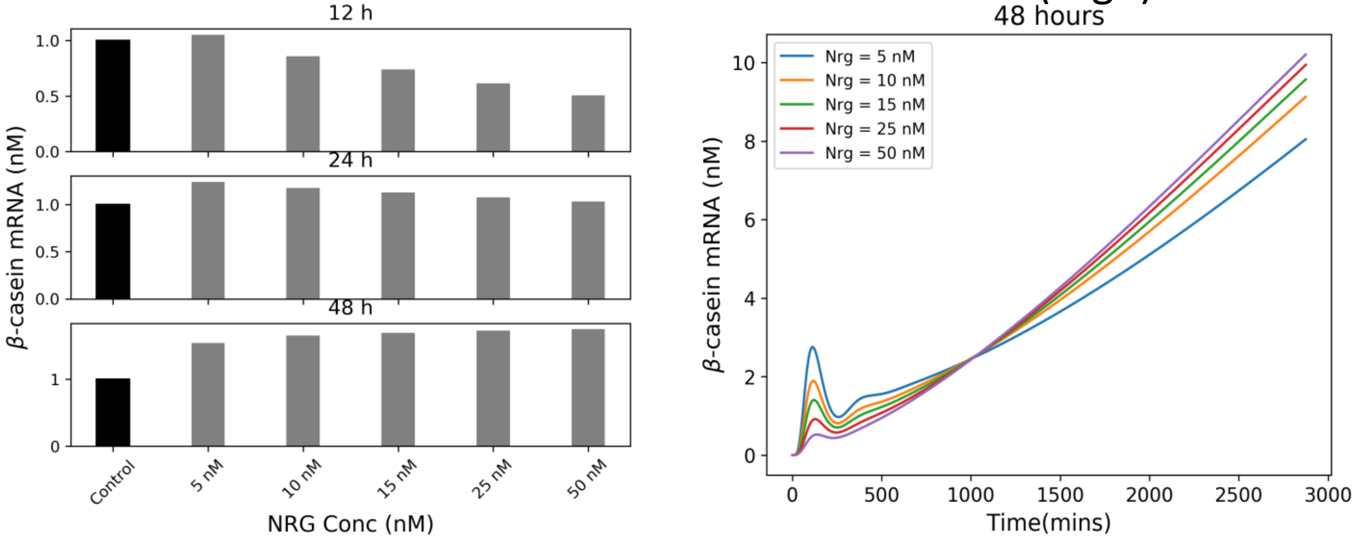

Figure 13

HER4-HER2 Heterodimerization (Low)

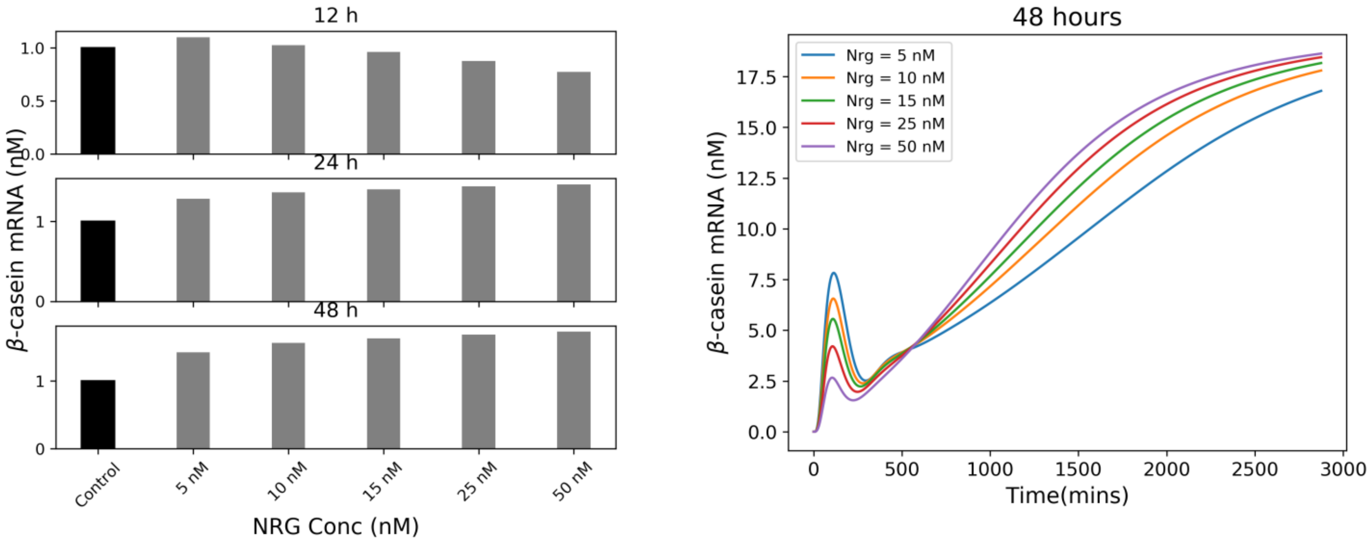

HER4-HER2 Heterodimerization (Medium)

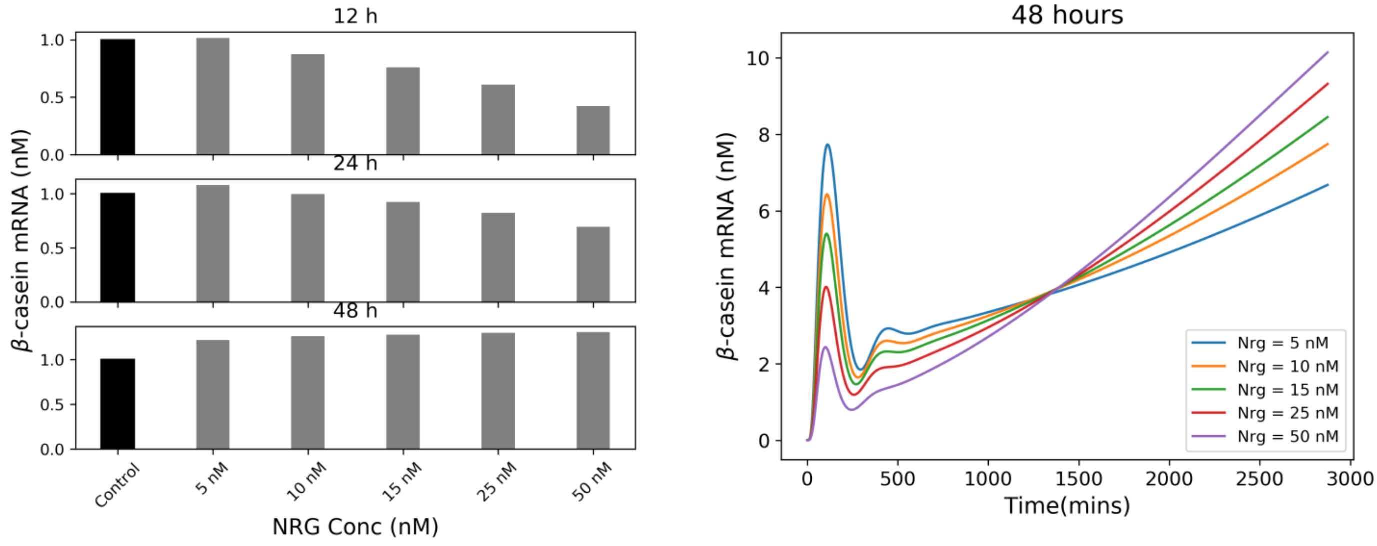

HER4-HER2 Heterodimerization (High)

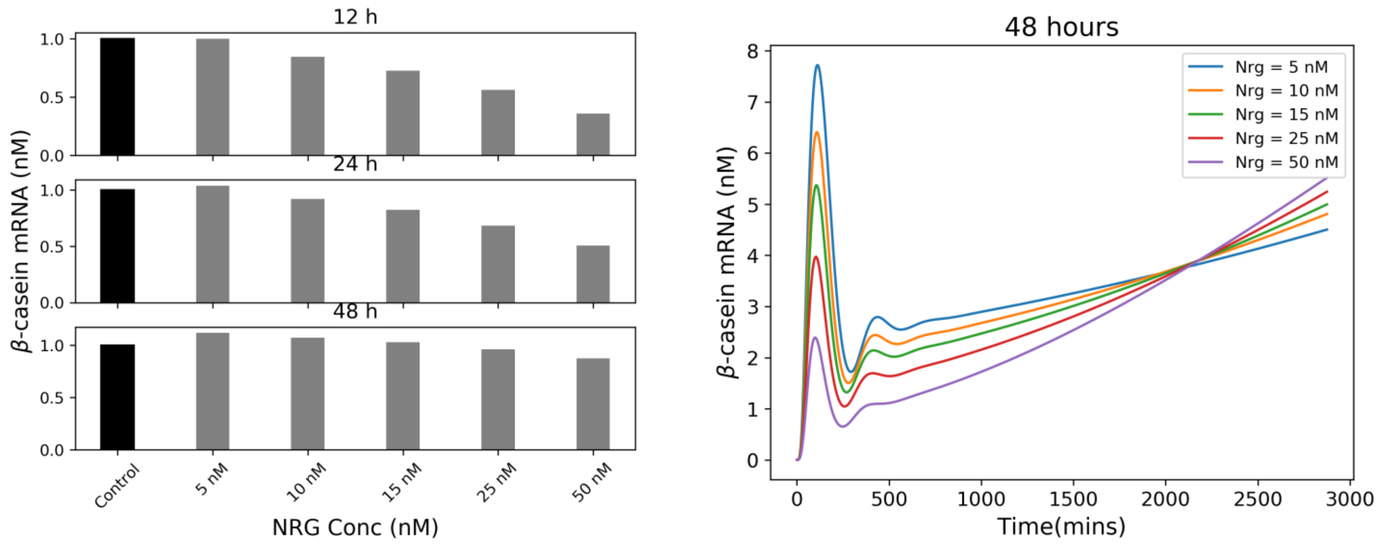

Figure 14

HER4 JAK-Independent Activation (Low)

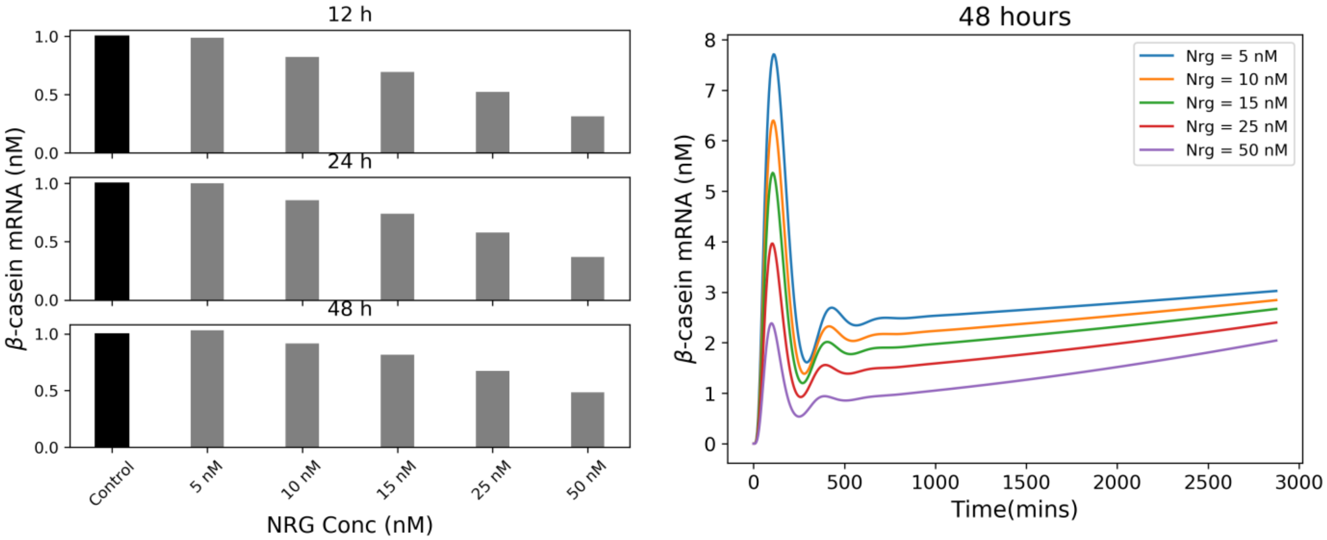

HER4 JAK-Independent Activation(Medium)

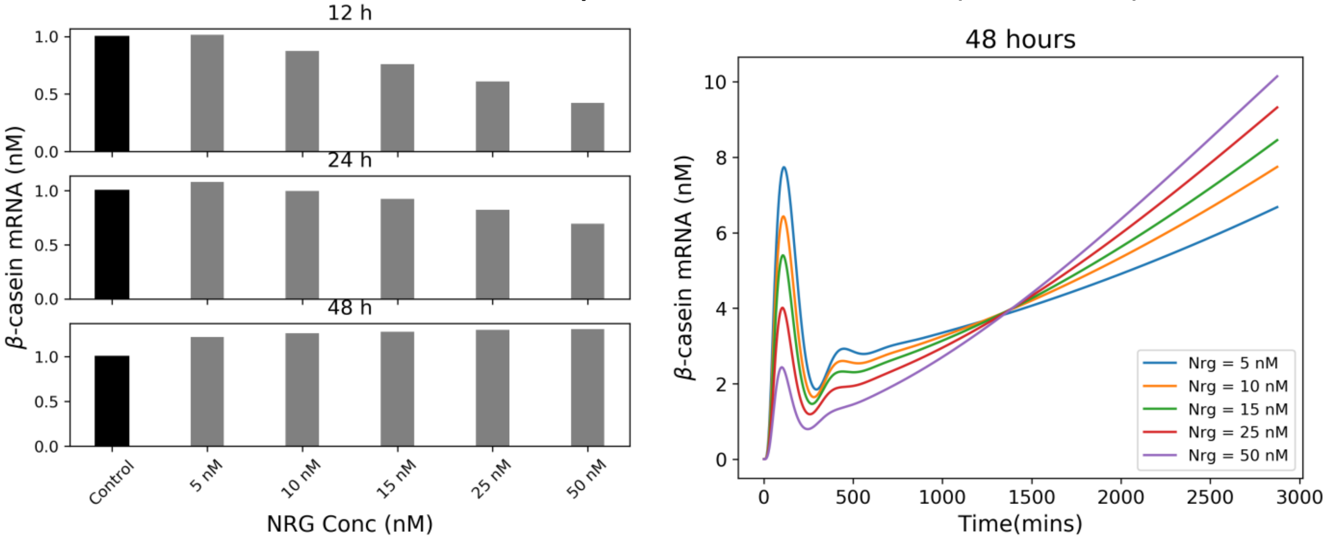

HER4 JAK-Independent Activation (High)

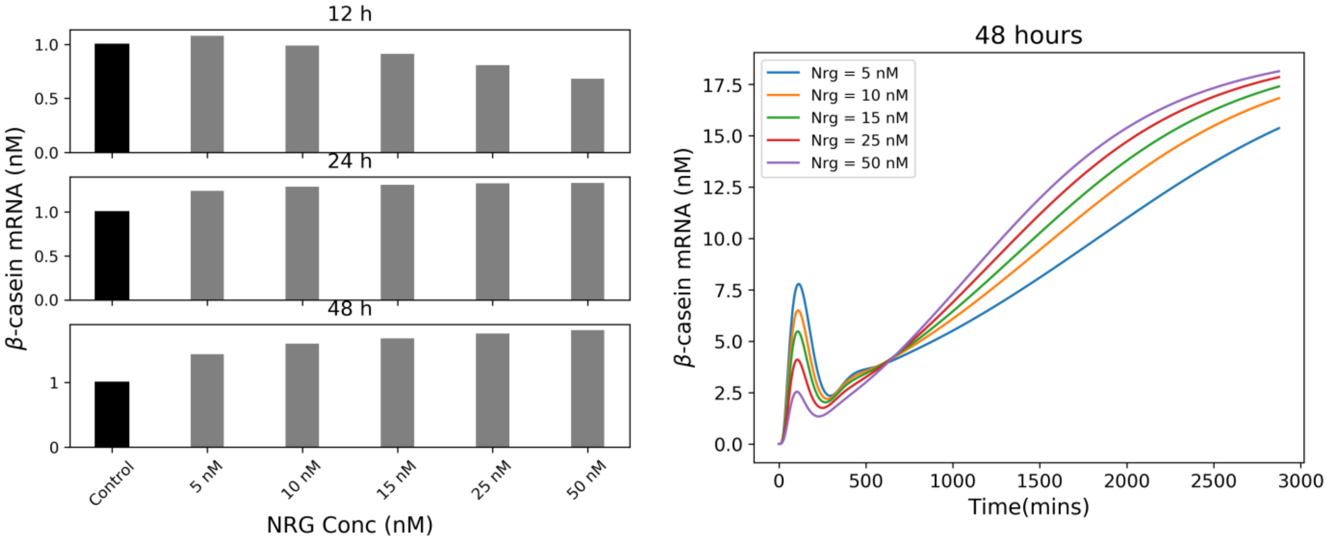

Supplementary Figure Captions

Figure 1: Sobol first order (S1) and total effect sensitivities (ST) for the HER4-JAK-STAT model at low (1 nM) NRG stimulations.

Figure 2: Sobol first order (S1) and total effect sensitivities (ST) for the HER4-JAK-STAT model at high (20 nM) NRG stimulations.

In these plots, we mostly see the same set of species and parameters as the NRG stimulation used (10 nM) in the main section. One difference is observed in the transcription delay where more effect of the transport rates is seen at 10 nM than at low NRG.

Figure 5-10: Parameter sweep plots for three different initial concentrations (low, nominal and high) of different species in the model. The low and high bounds were obtained by applying fixed percentages (15-20%) on the nominal set of values. Both time-integrated and instantaneous time profiles are shown. Species like HER2 and HER4 which were observed to be highly sensitive before were seen to alter the time profiles at different ligand stimulations drastically. They also influenced the time-dependent switching behavior by shifting both the peak mRNA values and also the times of transition.

Figure 11-14: Parameter sweep plots for three values (low, nominal and high) of different reactions in the model. The low and high bounds were obtained by applying fixed percentages (15-20%) on the nominal set of values. Both time-integrated and instantaneous time profiles are shown. The transcription Hill-coefficient greatly influenced both overall dynamics of transcription the switching behavior. The HER4 heterodimerization rates also changed the late time dynamics and transition times. These reactions show why HER4 and HER2 initial numbers were also sensitizing. Much of the influence of these receptors came through these dimerization reactions. The nuclear translocation rate of STAT5 dimer like mRNA export rate shown in figures in the main text influenced the delay and timing of switch. Finally, the JAK-independent HER4 activation also changed the late time behavior. At low activation, it produced a steady level of activation in 24-48 hour period while at high levels of activation it produced a substantial increase in the late stages.
